# *Pterodactylus scolopaciceps* Meyer, 1860 (Pterosauria, Pterodactyloidea) from the Upper Jurassic of Bavaria, Germany: The Problem of Cryptic Pterosaur Taxa in Early Ontogeny

**DOI:** 10.1371/journal.pone.0110646

**Published:** 2014-10-22

**Authors:** Steven U. Vidovic, David M. Martill

**Affiliations:** School of Earth and Environmental sciences, University of Portsmouth, Portsmouth, United Kingdom; University of Pennsylvania, United States of America

## Abstract

The taxonomy of the Late Jurassic pterodactyloid pterosaur *Pterodactylus scolopaciceps* Meyer, 1860 from the Solnhofen Limestone Formation of Bavaria, Germany is reviewed. Its nomenclatural history is long and complex, having been synonymised with both *P*. *kochi* (Wagner, 1837), and *P*. *antiquus* (Sömmerring, 1812). The majority of pterosaur species from the Solnhofen Limestone, including *P. scolopaciceps* are represented by juveniles. Consequently, specimens can appear remarkably similar due to juvenile characteristics detracting from taxonomic differences that are exaggerated in later ontogeny. Previous morphological and morphometric analyses have failed to separate species or even genera due to this problem, and as a result many species have been subsumed into a single taxon. A hypodigm for *P. scolopaciceps*, comprising of the holotype (BSP AS V 29 a/b) and material Broili referred to the taxon is described. *P*. *scolopaciceps* is found to be a valid taxon, but placement within *Pterodactylus* is inappropriate. Consequently, the new genus *Aerodactylus* is erected to accommodate it. *Aerodactylus* can be diagnosed on account of a unique suite of characters including jaws containing 16 teeth per-jaw, per-side, which are more sparsely distributed caudally and terminate rostral to the nasoantorbital fenestra; dorsal surface of the skull is subtly depressed rostral of the cranial table; rostrum very elongate (RI = ∼7), terminating in a point; orbits correspondingly low and elongate; elongate cervical vertebrae (approximately three times the length of their width); wing-metacarpal elongate, but still shorter than the ulna and first wing-phalanx; and pteroid approximately 65% of the total length of the ulna, straight and extremely thin (less than one third the width of the ulna). A cladistic analysis demonstrates that *Aerodactylus* is distinct from *Pterodactylus*, but close to *Cycnorhamphus* Seeley, 1870, *Ardeadactylus* Bennett, 2013a and *Aurorazhdarcho* Frey, Meyer and Tischlinger, 2011, consequently we erect the inclusive taxon Aurorazhdarchidae for their reception.

## Introduction

The genus *Pterodactylus* Cuvier 1809 [1] has a long and complex taxonomic history, and its definition and membership have changed considerably over time. Today the genus is well defined, but its taxonomic content remains contentious. Most recently, Bennett [2] includes only the type species *Pterodactylus antiquus* (Sömmerring, 1812) [3] within the genus, with which he synonymised *Pterodactylus kochi* (Wagner, 1837) [4]. Bennett [2] also synonymised *P. micronyx* Meyer, 1856 [5] with *Aurorazhdarcho primordius* Frey et al., 2011 [6] over which the species name *micronyx* has priority. He considered *Pterodactylus longicollum* Meyer, 1854 [7] to be generically distinct and placed it in the new genus *Ardeadactylus*. Several species of *Pterodactylus* had previously been synonymised with *P*. *kochi*, one of which, *Pterodactylus scolopaciceps* Meyer, 1860 [8] is discussed here.

**Figure 1:**
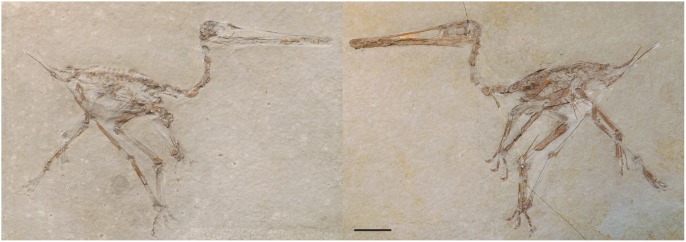
The part and counterpart of *Pterodactylus scolopaciceps* (BSP AS V 29 a [right] b [left]) holotype. Scale bar = 20 mm.

**Figure 2:**
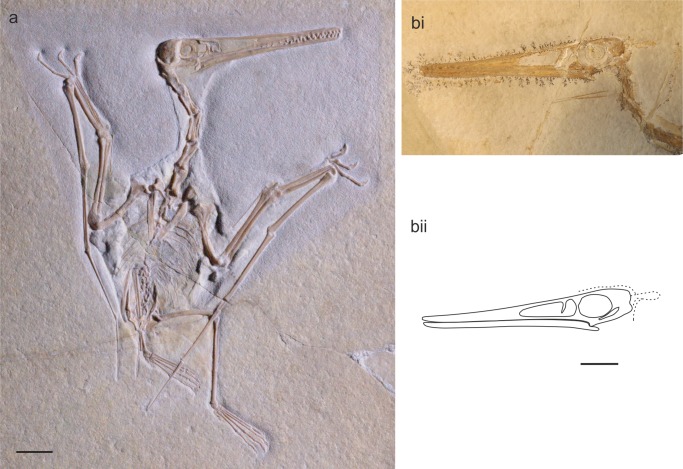
*Pterodactylus* specimens with soft tissue. A) Broili (1938) specimen of *Pterodactylus scolopaciceps* (BSP 1937 I 18) demonstrating extensive soft tissue (scale bar = 20 mm); B) soft tissue occipital head crest from a “*Pterodactylus kochi*” specimen (BSP 1883 XVI 1); i) photograph; ii) line drawing (scale bar = 20 mm).

*Pterodactylus scolopaciceps* is a species of pterodactyloid pterosaur that has for more than 130 years been regarded as a junior synonym of *Pterodactylus kochi*. The holotype (BSP AS V 29 a/b) (Fig. 1) and all other examples of *P*. *scolopaciceps* were discovered in the Solnhofen Limestone Formation (Late Jurassic, Tithonian) of Eichstätt, in northern Bavaria, Germany, giving a stratigraphic age of ∼152 ma [9]. The Solnhofen Limestone is a fine grained, lithographic limestone (plattenkalk), famous for the high quality of its fossil assemblage [10], [11], with an abundance of pterosaurs, sometimes with soft tissue preservation [12], [13], [14], [15], [16]. The majority of specimens described here are complete, or near complete articulated skeletons. One, BSP 1937 I 18 referred to *P. scolopaciceps* by Broili [17] preserves the soft tissue silhouette of the animal, demonstrating the integument about the neck and the wing membranes (Fig. 2a), while a number of other specimens preserve a soft tissue head crest (Fig. 2b). Consequently, *P. scolopaciceps* is anatomically one of the most informative pterodactyloids known.

**Figure 3:**
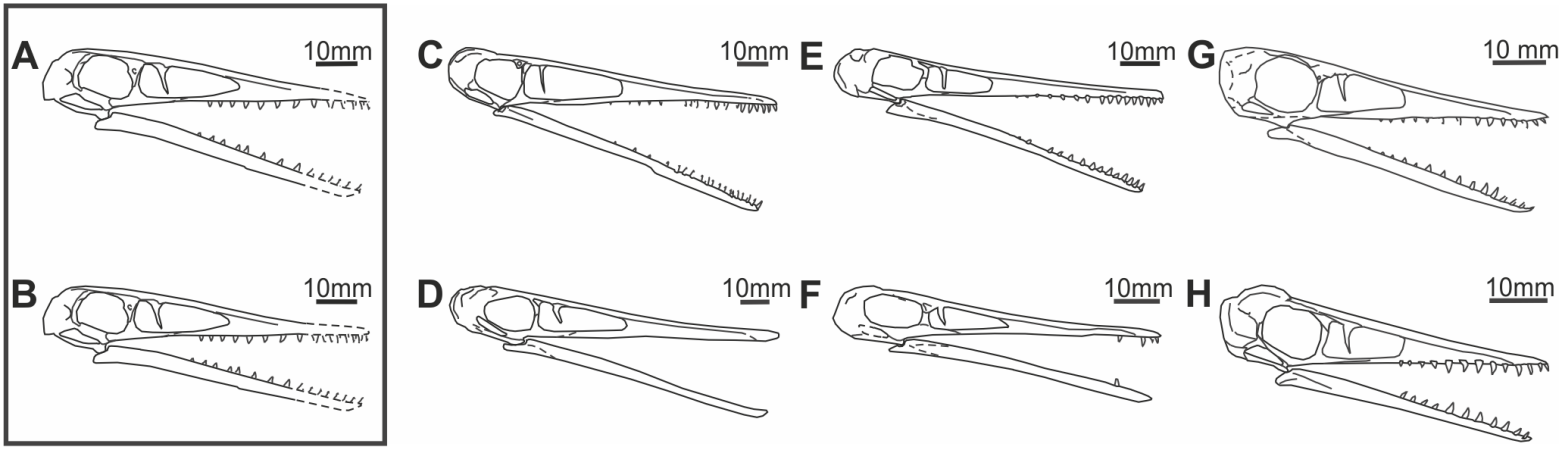
Line drawings of seven skulls belonging to *Pterodactylus antiquus* (*sensu* Bennett [2]). A & B) two opposing reconstructions of the holotype of *P. kochi* (SMF R 404/BSP AS XIX); C) the holotype of *Pterodactylus antiquus* (BSP AS I 739); D) a large specimen of “*Pterodactylus kochi*” (BSP 1883 XVI 1); E) Broili (1938) specimen of *Pterodactylus scolopaciceps* (BSP 1937 I 18); F) the holotype of *Pterodactylus scolopaciceps* (BSP AS V 29 a/b); G) a small juvenile specimen of *Pterodactylus kochi* (NHMUK PV R 3949, OUMNH JZ 1609); H) a small juvenile specimen of *Pterodactylus kochi* (SMF R 4072). Scale bars = 10 mm.

The taxon *Pterodactylus scolopaciceps* is often attributed to Meyer 1850 (e.g. [15]). However, this is an error, because Meyer did not erect *P*. *scolopaciceps* until 1860. In Meyer’s 1850 paper, he described BSP AS V 29 a/b as an example of *P*. *longirostris* Cuvier, 1819 [18], a name that is now regarded as a junior synonym of *P*. *antiquus* (Sömmerring, 1812) [3]. In his now classic *Fauna der Vorwelt*, Meyer (1845–1860) [8] recognised that BSP AS V 29 a/b was distinct from *P*. *longirostris* on account of the shape of the dorsal face of the rostrum, and the relative proportions of the wing bones. Accordingly he erected the new species *P*. *scolopaciceps* for its reception, the name reflecting its superficial similarity to the Eurasian Woodcock (*Scolopax rusticola*), a bird with a very long bill. Wagner (quoted by Zittel [14]) considered the diagnostic features observed in the skull of BSP AS V 29 a/b to be taphonomic artefacts, or even misinterpretations on Meyer’s part. Zittel [14] concluded that *P. scolopaciceps* was synonymous with *P. kochi* and further suggested that both could be synonymous with *Pterodactylus longirostris* ( = *P*. *antiquus*). Disregarding Zittel’s [14] synonymy, based on the graphs presented by Wiman [19] and the dorsal slope of the skull, Broili [17] referred a new pterosaur specimen (BSP 1937 I 18) from Eichstätt to *P. scolopaciceps* (Fig. 2a & Fig. 3e). Later, Wellnhofer [15] re-examined the specimen, referring it to *P. kochi*, thus accepting Zittel’s [14] synonymy. Subsequent specimens displaying similar morphologies to BSP AS V 29 a/b and BSP 1937 I 18 have also been referred to *P. kochi* (e.g. the specimen of Frey and Martill [20]).

The taxonomic status of *Pterodactylus kochi* and its contents received some attention in the late 20^th^ and early 21^st^ Century [21], [22], [23], [24]. Bennett [2] synonymized *P. kochi* (Fig. 3a, b) with *Pterodactylus antiquus* (Fig. 3c), following Zittel’s (1883) suggestion. Bennett [2] performed his synonymy on the basis of Nopcsa curves, principal component analyses and the lack of detectable morphological characteristics distinguishing it from *P. antiquus*. However, Bennett [2] noted that the holotype of *P. kochi* was from Kelheim, where the stone quarries are stratigraphically younger than those of Eichstätt, where specimens of *P. scolopaciceps* come from.

Synonymy between *P. antiquus* and *P. kochi* had been suggested several times [14], [21], [22], [24] prior to Bennett’s [2] comprehensive study and definitive synonymization of the two. Despite the concerns raised over the validity of *P. kochi* and the inherent taxonomic instability, the taxon was coded in several cladistic analyses (e.g. [25], [26]) without discussion. Furthermore, the taphonomic condition of the *P. kochi* holotype cross-referenced with the completeness of coding in these analyses, suggests that one of the referred specimens, most likely pertaining to *P. scolopaciceps* was coded instead (the authors did not specify).

A study of specimens referred to *P. kochi* by one of the authors (SUV) revealed that the cervical vertebrae of some specimens appear more elongate than those of the holotype (SMF R 404/BSP AS XIX). Furthermore, those specimens with elongate cervical vertebrae appeared to possess a more elongate skull with a slight depression in its dorsal surface. Included among these distinctive specimens is BSP AS V 29 a/b, the holotype of *P. scolopaciceps.* Although Wagner (in [14]) and Zittel [14] considered the skull’s dorsal depression to be a taphonomic artefact, the number of specimens displaying this morphology suggest that it is an original anatomical feature (e.g. BSP 1937 I 18). Consequently, specimens referred to *P. scolopaciceps* are reappraised.

## Materials and Methods

Specimens in the following institutions were examined (note that no permits were required for the described study, which complied with all relevant regulations): Bayerische Staatssamlung für Paläontologie Munich, BSP; Natural History Museum, London, NHMUK; Naturhistorisches Museum Basel, NMB; Oxford University Museum of Natural History, OUMNH; Senckenberg Museum, Frankfurt, SMF. In addition we used published images of pterosaurs for supplementary morphological data from a variety of referenced sources, but especially Wellnhofer [15]. Additional institutional abbreviations: Institute of Geology, Chinese Academy of Geological Sciences, Beijing, IG-CAGS; Museum of Comparative Zoology, Harvard, Massachusetts, MCZ; Naturhistorisches Museum Wien, Vienna, NHMW.

**Figure 4:**
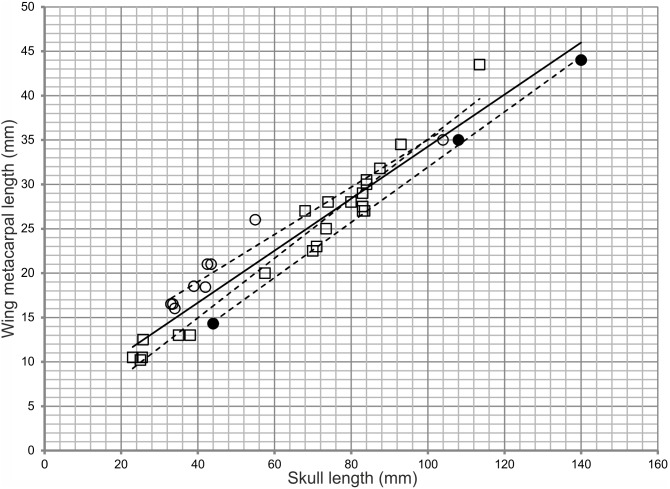
A graph of Solnhofen pterodactyloids compiled from data available in Wellnhofer [15]. Skull length plotted along the x-axis, wing metacarpal length plotted along the y-axis. Despite clear morphological differences a crowded data set has resulted in all specimens appearing similar. A similar situation is observed when all other elements plotted against skull length. *P. antiquus* filled circles, *P. kochi* open squares, *Ctenochasma* open circles. R^2^ values: all specimens = 0.931; *P. antiquus* specimens = 0.998; *P. kochi* specimens = 0.959; *Ctenochasma gracile* + *P. elegans* ( = *Ctenochasma elegans*) = 0.942. Dashed lines are regression lines for each respective taxon, the solid line is the regression line for all specimens.

In a preliminary morphometric analysis we plotted data for *P. kochi* and *P.antiquus* taken from Wellnhofer’s [15] study, and incorporated data from *Ctenochasma elegans* as a comparator, in order to demonstrate the spread of data between morphologically distinct specimens (Fig. 4). The results of this preliminary analysis (see File S2) revealed little or no disparity between taxa despite the inclusion of the morphologically distinct *Ctenochasma*. To avoid misinterpretations based on crowded datasets we then performed a focused study that included the holotypes of *Pterodactylus antiquus* (BSP AS I 739), *P. scolopaciceps* (BSP AS V 29 a/b) and *P. kochi* (SMF R 404/BSP AS XIX), four specimens (BSP 1937 I 18, BSP 1883 XVI 1 [Fig. 3d], BSP 1975 I 221, SMF R 4072 [Fig. 3h]) previously referred to *P. kochi* [15] and a specimen that has been labelled *P*. *kochi* in the NHMUK collection (NHMUK PV R 3949/counterpart OUMNH JZ 1609) (Fig. 3g). As with most pterosaur specimens from the Bavarian plattenkalks, all specimens studied here are sub-adult [22], demonstrating skull lengths between 56 and 112 mm. We undertook a series of morphometric analyses and statistical tests (e.g. using trend lines and their respective R^2^ values to perform analyses of covariance [ANCOVA], and an honestly significant difference test [HSD]), including the plotting of scatter graphs for 16 linear measurements of pterosaur anatomy using Microscoft Excel. The 16 linear measurements have proven useful in distinguishing taxa in cladistics analyses and differential diagnoses, and are preserved in the majority of specimens studied here. To avoid the “Texas sharp shooter fallacy” [27] we constructed two hypothetical taxa: morphotypes one and two. The two morphotypes were based on anatomical differences between specimens of *P. antiquus* (*sensu* Bennett [2]), that were observed during data collection for a comprehensive phylogenetic review. Morphotype one (SMF R 404/BSP AS XIX, SMF R 4072, NHMUK PV R 3949/OUMNH JZ 1609) consisted of all specimens previously referred to *P. kochi* possessing a straight rostrum and relatively short cervical vertebrae compared to the *P. antiquus* holotype. Morphotype two (BSP AS V 29 a/b, BSP 1937 I 18, BSP 1883 XVI 1, BSP 1975 I 221) consisted of the remainder of specimens referred to *P. kochi*, all of which possess an elongate, depressed rostrum and have elongate cervical vertebrae. *Pterodactylus antiquus* was analysed independently as it did not fit into either of these morphotypes. In addition to analysing the two morphotypes and *P. antiquus* individually, all specimens were studied together ( = *P. antiquus* sensu Bennett [2]) to compare R^2^ values for the *P. antiquus* hypodigm to each respective morphotype.

The validity of these hypothetical taxa was tested using linear measurements of the skull, wing, leg and vertebral series, which were compared using scatter graphs. Sixteen direct measurements were taken from the eight specimens (see File S2), but in the case of SMF R 404/BSP AS XIX the skull length was estimated by projecting the dorsal and ventral margins of the rostrum to the point where they intersected. A skull length proxy (caudal margin of the squamosal to the rostral margin of the nasoantorbital fenestra) was also employed to assess the accuracy of the estimated skull length. Ideally, a discriminant function analysis would have been used to test the validity of the morphotypes, but limitations due to the number of specimens available made this impractical. Instead, each measurement was compared to all others, generating 120 bivariate graphs (see File S2). Confidence ellipses [27] (CHI = 2.45, resulting in a 95% confidence limit) were calculated for each morphotype in the figured graphs to demonstrate statistically significant data separation. When two regression lines on a single graph were demonstrating similar slopes to each other ANCOVA was performed. To perform ANCOVA each regression line was projected to intersect the y-axis ( = 0 on x-axis). If the two regression lines were distinct where they intersected the y-axis, this difference was considered significant and independent of the size variation in the sample. To consolidate the results from the 120 graphs, the frequencies of R^2^ values (using intervals of 0.01) for each of the morphotypes and the all-inclusive sample (i.e. *Pterodactylus antiquus* sensu Bennett [2]) were plotted onto a line diagram. Subsequently, a Tukey-Kramer HSD test [27] was performed on the three datasets of R^2^ values (see File S2). For each combination of tests (i.e. morphotype one *vs* morphotype two; morphotype two *vs* all-inclusive sample etc.) q_s_ was calculated and compared to the HSD number of the entire study group. Where q_s_ is greater than HSD the separation is statistically significant.
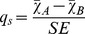


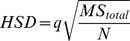



**Figure 5:**
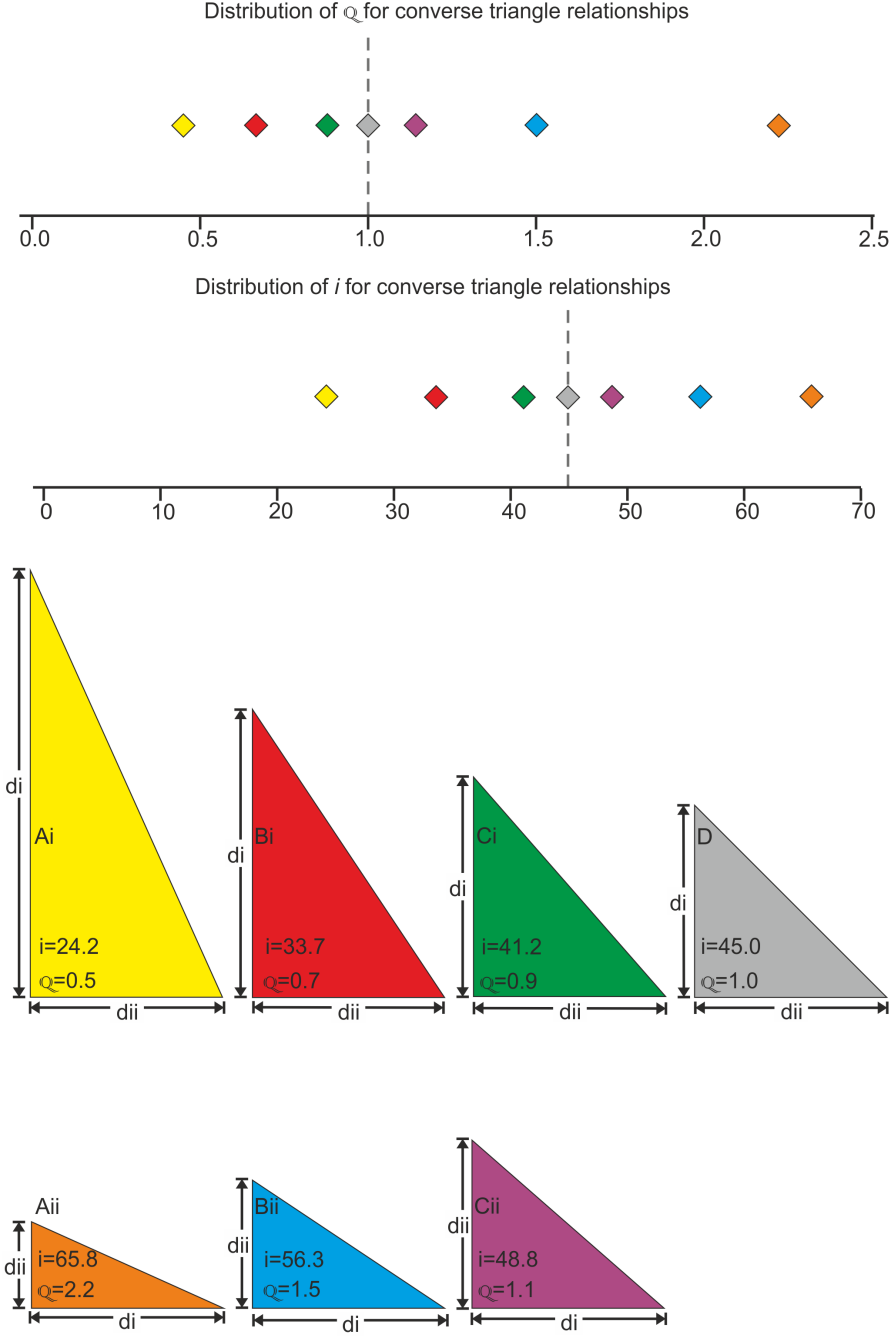
A demonstration of *i = tan^−1^a/b* rescaling on triangles with opposing dimensions. The plots at the top demonstrate the data distribution of the triangles illustrated below, where Q = raw quotient value of triangle length/depth, and *i* = rescaled quotient values of triangle length/depth. The colours of triangles Ai – Ci, Aii – Cii and D are replicated in the diamonds in the plots for clarity. The depths (di) of Ai – Ci are equal to the lengths (also di) of Aii – Cii respectively. Likewise, the lengths (dii) of Ai – Ci are equal to the depths (also dii) of Aii – Cii. D is an equilateral triangle, and on the plots it demonstrates the transition between tall and long forms. The diagram demonstrates that triangles with the same range of variation between both shallow-long forms and deep-short forms will only exhibit the true range of variation once the *i = tan^−1^a/b* rescaling has been applied.

A cladistic analysis of the Pterodactyloidea, including all of the taxa discussed here was also performed. The analysis included 31 pterodactyloids and two non-pterodactyloid monofenestratans as an outgroup. The taxa were coded for 127 characters, of which 61 are cranial and 66 are postcranial. Ten characters have continuous states, the analysis of which was made possible by employing TNT’s “new technology search” [28]. Four of the continuous characters required rescaling with the equation *i = tan^−1^a/b*, where *i* is the index number analysed and *a/b* is the quotient value of the elements being studied. It was necessary to apply this equation to characters with converse data in their coding *i.e.* where the ratios between elements are reversed between different specimens. By applying this rescaling equation, directly inverse data points have a symmetrical distribution on a linear graph, avoiding mathematical bias (Fig. 5). Additionally, many characters from previous analyses were split into their component parts to avoid the undesirable effect of compound characters on the resulting MPTs (most parsimonious trees) [29]. In some cases compound characters were deleted, with new characters formulated to replace them. In total there are 75 new or considerably modified characters in the new analysis.

**Figure 6:**
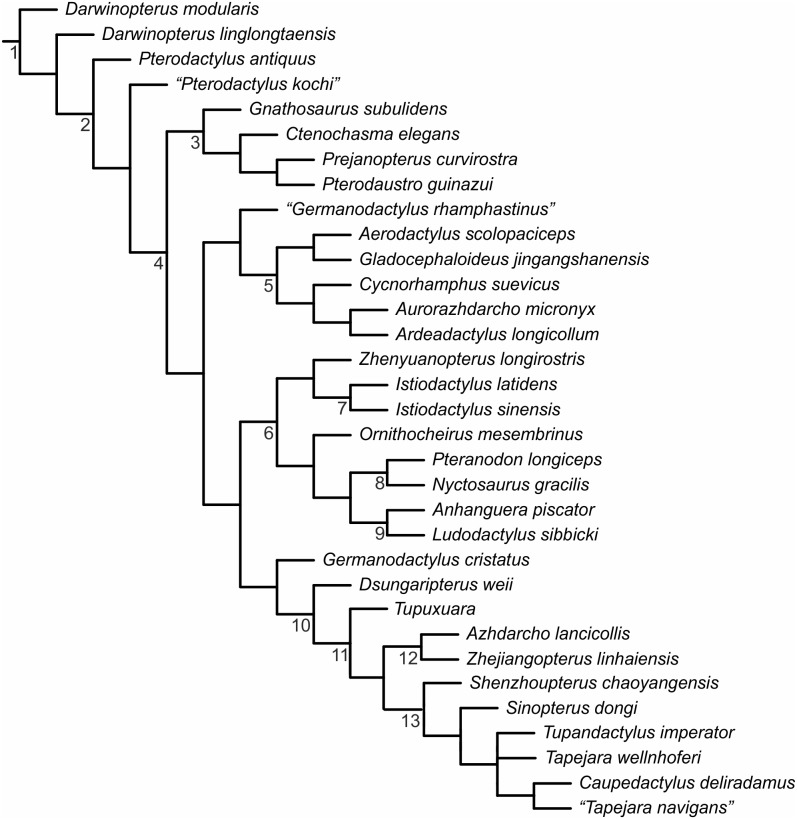
A single most parsimonious tree of the Pterodactyloidea recovered using a TNT “new technology search”. Named nodes: 1 = Monofenestrata Lü et al. 2010 [30]; 2 = Pterodactyloidea Plieninger 1901 [31]; 3 = Ctenochasmatidae Nopcsa 1928 [32]; 4 = Lophocratia Unwin 2003 [33]; 5 = Aurorazhdarchidae *fam. nov.*; 6 = Ornithocheiroidea Seeley 1891 [34]; 7 = Istiodactylidae Howse et al. 2001 [35]; 8 = Pteranodontia Marsh 1876 [36]; 9 = Anhangueridae Campos and Kellner 1985 [37]; 10 = Tapejaroidea Kellner 1996 [38]; 11 = Azhdarchoidea Nesov 1984 [39]; 12 = Azhdarchidae Nesov 1984 [39]; 13 = Tapejaridae Kellner 1989 [40].

Due to missing data from taphonomic effects and under-sampling all discrete characters were treated as unordered with equal weights. The tree (Fig. 6) was generated using a “new technology search” in TNT. A further traditional search using TBR was run on the trees stored to RAM, to ensure that the maximum MPTs were recovered. Full details are provided in File S1.

### Nomenclatural acts

The electronic edition of this article conforms to the requirements of the amended International Code of Zoological Nomenclature, and hence the new names contained herein are available under that Code from the electronic edition of this article. This published work and the nomenclatural acts it contains have been registered in ZooBank, the online registration system for the ICZN. The ZooBank LSIDs (Life Science Identifiers) can be resolved and the associated information viewed through any standard web browser by appending the LSID to the prefix “http://zoobank.org/”. The LSID for this publication is: urn:lsid:zoobank.org:pub:FF00F1B7-1C8F-497C-BC3D-CC866A5850BB. The electronic edition of this work was published in a journal with an ISSN, and has been archived and is available from the following digital repositories: PubMed Central, LOCKSS.

## Results

### Graphical analyses

Scatter plots of Wellnhofer’s [15] measurements of *Pterodactylus* and *Ctenochasma* (File S2) demonstrate similarity between all specimens, despite clear morphological differences [24]. The graph of skull length *vs* wing metacarpal length has an R^2^ value of 0.931 for all specimens (Fig. 4). Specimens referred to *Pterodactylus antiquus* cluster tightly to the regression line, whereas specimens referred to *P. kochi* are more disparate, but regardless of the spread of data the taxa are well supported by the R^2^ values. When examining individual specimens referred to *P*. *antiquus* by Bennett [2], it is clear that substantial morphological differences exist between them. For example, a specimen Bennett regards as *P*. *antiquus*, but identified as *P. kochi* (BSP 1883 XVI 1) by Wellnhofer [15] has a skull length ∼26 mm (23% of total skull length) shorter than another specimen (RM St. 18 184) Wellnhofer [15] referred to *P. antiquus* despite a similar wing metacarpal length for both (Fig. 4). Although such a difference might reflect ontogenetic variation, the similar size of the metacarpal suggests otherwise. Intraspecific variation might also account for any variation, but such significant differences have not previously been reported for the Pterosauria, thus more likely reflecting a taxonomic distinction.

One hundred and twenty graphs plotting the proportional differences between 16 linear measurements of the skeleton were analysed. Three different distributions of data were considered to be informative: 1) at least one strongly supported morphotype with a wide disparity between the two regression lines of each morphotype; 2) the trend lines of both morphotypes diverge or converge; 3) all specimens plot close to a single regression line (note the latter does not support the hypothesis).

**Figure 7:**
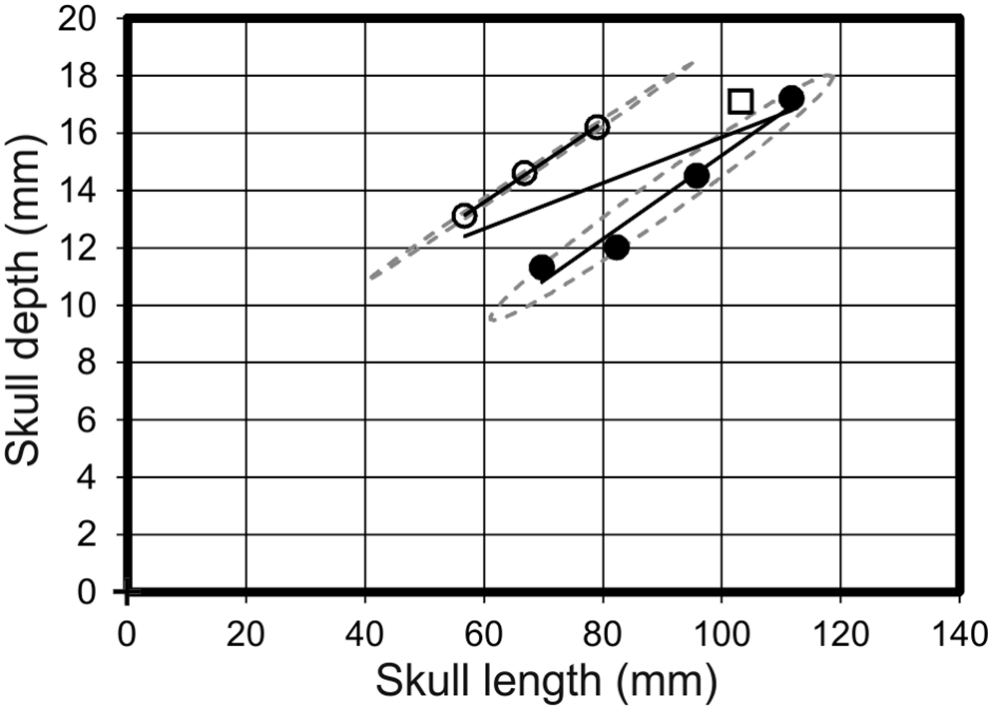
Skull length *vs* skull depth. A graph demonstrating the spread of data between morphotype one (open circles), morphotype two (filled circles) and *Pterodactylus antiquus* (Open square) in respect to their skull length and skull depth. Solid black lines = regression lines for respective morphotypes; dashed grey lines = 95% confidence limits.

**Figure 8:**
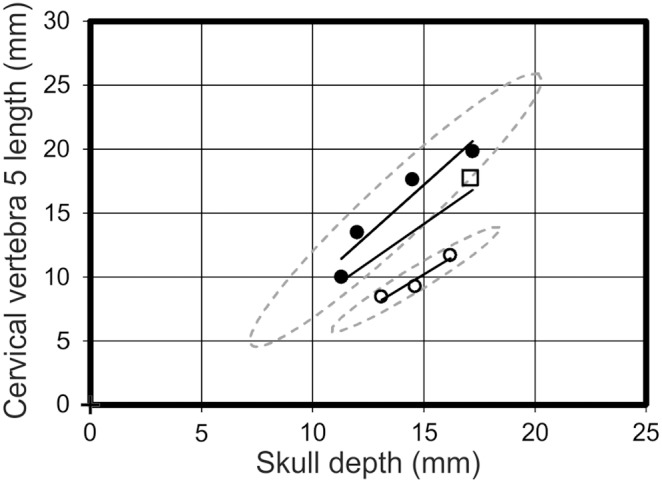
Skull depth *vs* cervical vertebra 5 length. A graph demonstrating the spread of data between morphotype one (open circles), morphotype two (filled circles) and *Pterodactylus antiquus* (Open square) in respect to their skull depth and cervical vertebra 5 length. Solid black lines = regression lines for respective morphotypes; dashed grey lines = 95% confidence limits.

**Figure 9:**
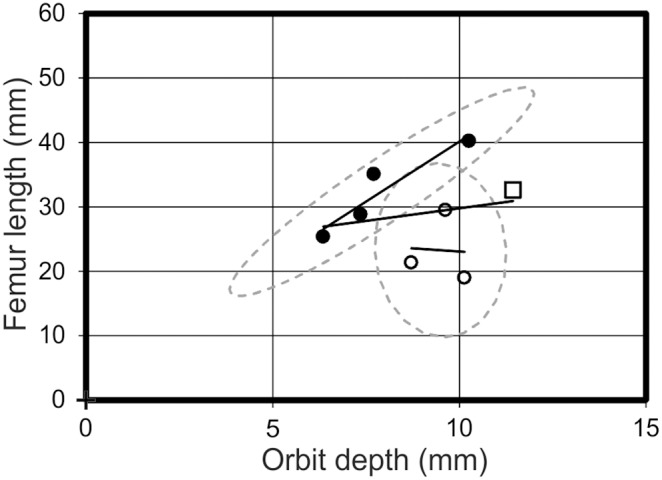
Orbit depth *vs* femur length. A graph demonstrating the spread of data between morphotype one (open circles), morphotype two (filled circles) and *Pterodactylus antiquus* (Open square) in respect to their orbit depth and femur length. Solid black lines = regression lines for respective morphotypes; dashed grey lines = 95% confidence limits.

A strongly supported morphotype with a wide disparity between the two regression lines (distribution 1 above) would report at least one strong R^2^ value for a morphotype, but a low value when all the specimens are analysed together. Notable examples include skull length *vs* skull depth (Fig. 7), skull depth *vs* humeral length, skull depth *vs* PCRW length (*praecaudale Rumpfwirbelsäule* = combined length of the dorsal and sacral vertebrae [2]), skull depth *vs* cervical vertebra five length (Fig. 8), and orbit depth *vs* femur length (Fig. 9). The 95% confidence ellipses for each morphotype contain 100% of specimens for each respective morphotype and graph. The confidence ellipses for skull length *vs* skull depth (Fig. 7) and skull depth *vs* cervical vertebra five length (Fig. 8) are well separated, but in the case of orbit depth *vs* femur length, a poorly supported relationship for morphotype one results in intersecting confidence ellipses. Additionally, when the regression lines on many of these graphs are projected to intersect the y-axis (ANCOVA), there is still notable separation between many of the regression lines. For example, orbit length *vs* orbit depth; skull length *vs* skull depth; and pes length *vs* PCRW all demonstrate covariance in the ANCOVA.

**Figure 10:**
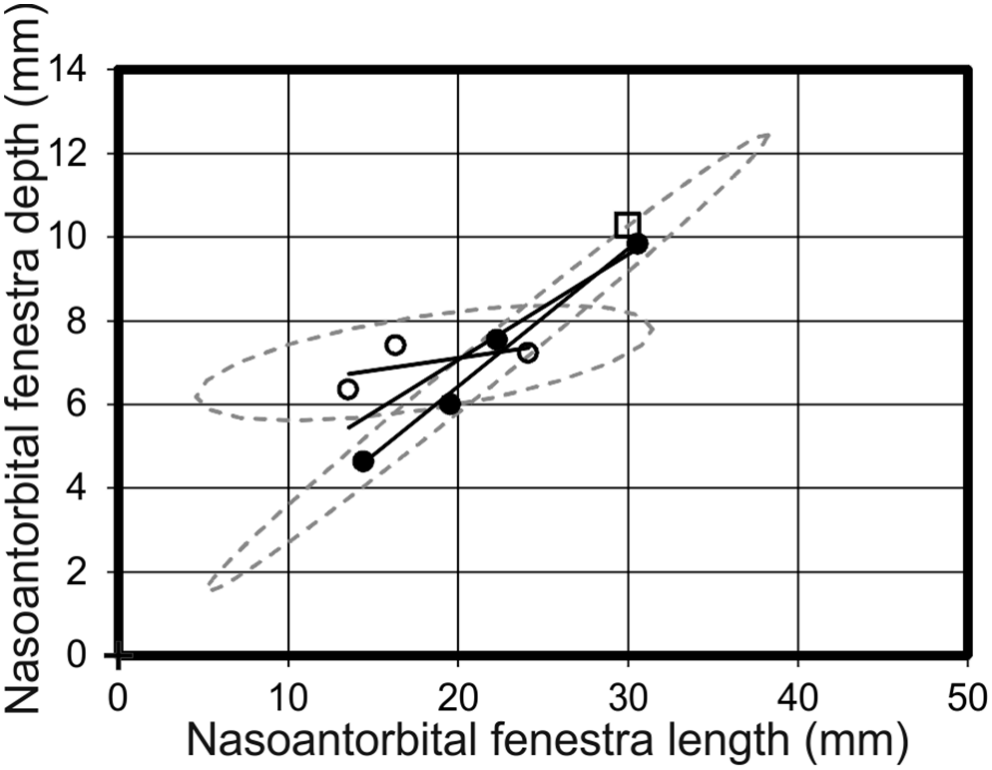
Nasoantorbital fenestra length *vs* nasoantorbital fenestra depth. A graph analysing the relationships of nasoantorbital fenestra length and depth dimensions, which demonstrates intersecting regression lines for morphotype one (open circles) and morphotype two (filled circles), *Pterodactylus antiquus* (open square) lies closest to morphotype two. Solid black lines = regression lines for respective morphotypes; dashed grey lines = 95% confidence limits.

**Figure 11:**
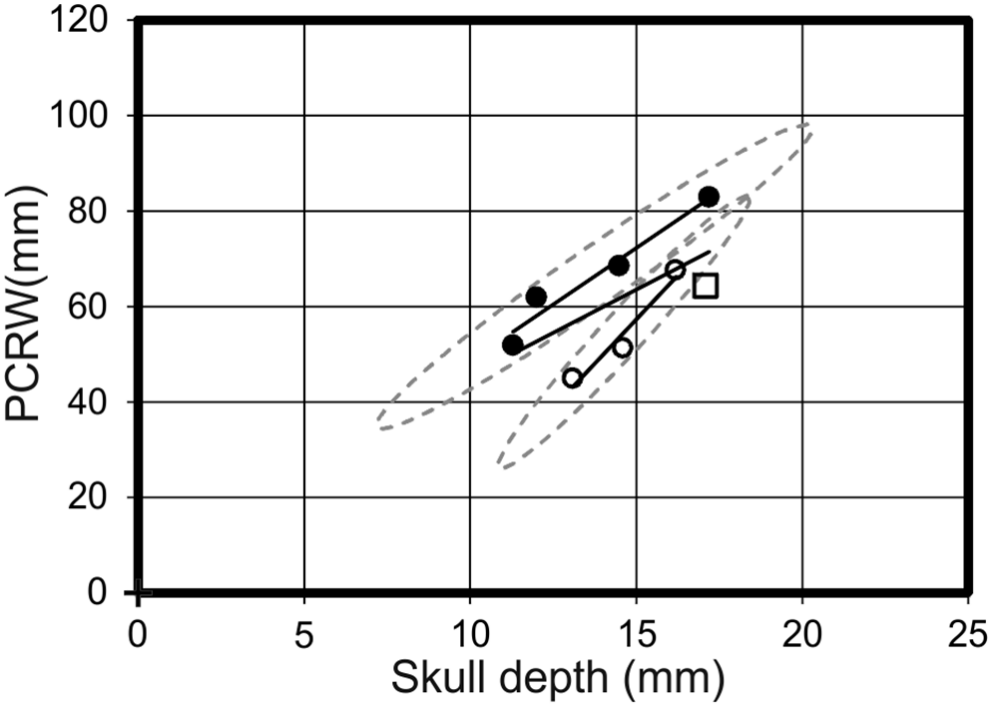
Skull depth *vs* PCRW. A graph demonstrating the spread of data between morphotype one (open circles), morphotype two (filled circles) and *Pterodactylus antiquus* (open square) in respect to their skull depth and the length of their PCRW. Solid black lines = regression lines for respective morphotypes; dashed grey lines = 95% confidence limits.

Distribution 2 would not necessarily be apparent in the R^2^ values, but clear differences between morphotypes are apparent on the graphs. An analysis of nasoantorbital fenestra length *vs* depth (Fig. 10) exhibits a cross-cutting relationship, with R^2^ values of 0.34 for morphotype one, 0.99 for morphotype two, and 0.77 for all specimens collectively. Despite the intersecting regression lines in the nasoantorbital fenestra length *vs* depth graph, two specimens of each morphotype lie outside of the other’s 95% confidence ellipse. Pes length *vs* humeral length demonstrates diverging regression lines, with R^2^ values of 0.99 for morphotype one, 0.98 for morphotype two, and 0.53 for all specimens collectively. The trend lines for skull depth *vs* cervical vertebra five length and PCRW both diverge to varying degrees. However, while skull depth *vs* cervical vertebra five length demonstrates a stronger positive allometry for morphotype two (Fig. 8), the PCRW demonstrates a stronger positive allometry for morphotype one (Fig. 11). In this case morphotype two has a much longer neck in later ontogeny if it represents a valid taxon, while ontogenetically less mature specimens of the two morphotypes are much more similar. Likewise, nasoantorbital fenestra length *vs* cervical vertebra five length and humeral length demonstrate strong support for each morphotype, but the diverging lines suggest dramatically different proportions would be present in later ontogeny.

For distribution 3 the hypothetical morphotypes have similar regression line slopes, that lie in the same locus on the graph, and the data plot tightly - regression lines for the data as a whole obviously show a relationship between all specimens. For many of the graphs demonstrating distribution 3 a specimen of *Ctenochasma elegans* (BSP 1935 I 24) was included in the analysis to further test the relationships. For example, when distance between the caudal extent of the squamosal to the rostral extent of the nasoantorbital fenestra was analysed against ulna length, strong support for each morphotype as well as all specimens was apparent. When the morphologically distinct *Ctenochasma* was also analysed R^2^ values still supported a relationship, demonstrating that the relationships observed operate at a higher taxonomic level and can be disregarded in the context of this study. The only parameters where this was not the case were those analysing the wing metacarpal, for which *Ctenochasma* lies in the extreme range as it possesses a highly elongate wing metacarpal. However, only five of the parameters analysed against the wing metacarpal suggested a relationship between all specimens of *P. kochi*, whilst the remainder (n = 10) exhibited distributions 1 and 2.

*Pterodactylus antiquus* was analysed with the specimens referred to *P. kochi* in order to test Bennett’s [2] species hypodigm, for which we find only a relatively poor signal of relationship (see above). In many of the 120 graphs analysing the linear measurements, *P. antiquus* is found to be closer to one or the other morphotype, but is not found to be closer to one particular morphotype at any notable frequency. For example, *P. antiquus* falls closer to specimens of morphotype two for characteristics of the cervical vertebrae, but closer to morphotype one for the PCRW length. For two out of the five graphs figured (Fig. 8 and 10) *P. antiquus* is located on the limit of the 95% confidence ellipses for morphotype two, while in all other tests it is a clear outlier.

**Figure 12:**
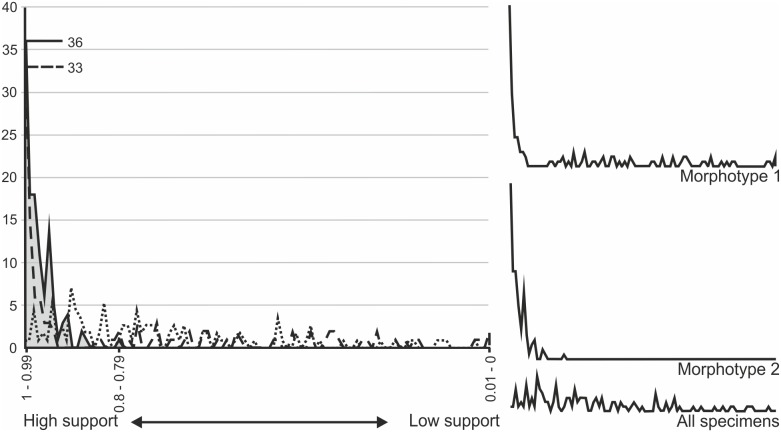
Absolute frequencies of R^2^ values from the extensive graphical analyses. A line graph demonstrating the frequencies (y-axis) of R^2^ values for each respective hypothesis tested in the 120 graphs. The R^2^ values are ordered from strong support to low support, left to right (x-axis). Morphotype one is represented by a dashed line, morphotype two is represented by a solid line, all specimens of “*Pterodactylus*” are represented by a dotted line. The highest peaks of the two morphotypes are marked and labelled. The area under the strongest supported hypothesis (morphotype two) is shaded grey for clarity. Likewise, the topologies of the lines are reproduced to the right of the graph.

The absolute frequencies of R^2^ values for each of the 120 graphs were plotted on a line graph (Fig. 12) to demonstrate the relative support for each hypothetical morphotype and all specimens (i.e. *Pterodactylus antiquus* hypodigm *sensu* Bennett [2]). The line graph demonstrates that both morphotype one (65% R^2^ values >0.75) and two (100% R^2^ values >0.75) are strongly supported when compared to the results from all the specimens analysed collectively (54% R^2^ values >0.75). However, morphotype two is significantly better supported than morphotype one. For morphotype two 96.7% of all R^2^ values are above 0.9, and all remaining R^2^ value support is greater than 0.79, compared to morphotype one with only 56.7% of all R^2^ values above 0.9, and as much as 20% below 0.5. The most striking results are orbit depth *vs* femur length (Fig. 9) and cervical vertebra five width, where morphotype one has an R^2^ value less than 0.1, suggesting that hypothetical morphotype one might contain more than one species. This is further supported by the Tukey-Kramer HSD test that shows no statistical significance between morphotype one and the all-inclusive sample, but supports a statistically significant difference in morphotype two with respect to morphotype one and the all-inclusive sample.

### Cladistic analysis

**Figure 13:**
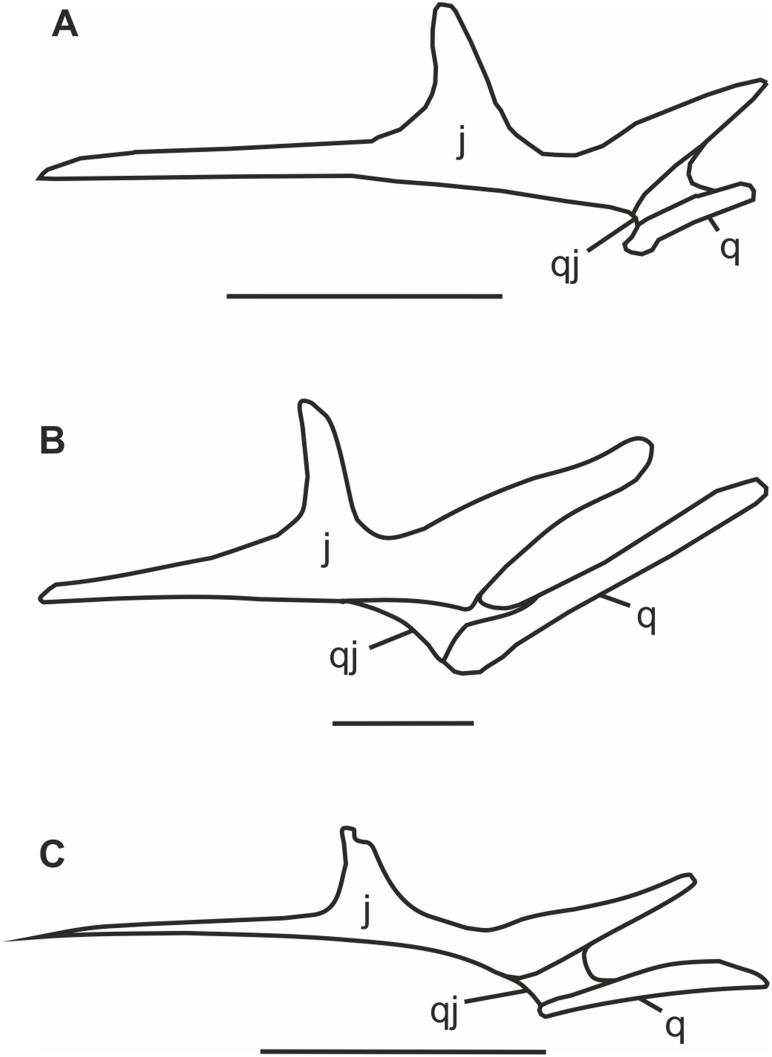
A demonstration of the breadth of the quadratojugal’s rostral portion. A) *Pterodactyulus antiquus*; B) *Cycnorhamphus suevicus*; C) *Aerodactylus scolopaciceps*. j, jugal; q, quadrate; qj, quadratojugal. Scale bars = 10 mm.

We performed a cladistic analysis using TNT to test the relationship between *Pterodactylus scolopaciceps*, 31 other pterodatyloid pterosaurs and two non-pterodactyloid monofenestratans, *Darwinopterus modularis* and *D*. *linglongtaensis* which were used as outgroup taxa. This analysis recovered a single tree with a length of 409.145 steps, with a radically different topology from that of most published pterodactyloid phylogenies (e.g. phylogenies of Unwin [33]; Kellner [26]; Wang et al. [41]; Lü *et al*. [30]; Andres & Myers [42]), although similarities were found with the analysis of Howse [43]. The Ctenochasmatoidea of Unwin [33] and the Archaeopterodactyloidea of Kellner [40], [26] are found to be polyphyletic, with their traditional contents occupying the basal part of the pterodactyloid tree as a series of metataxa or as members of distinct clades. The controversial *Germanodactylus* species are not congeneric, as suggested by Maisch *et al*. [44]. The type species *G. cristatus* is the sister taxon to Dsungaripteridae + Azhdarchoidea, while “*G. rhamphastinus*” lies at the base of a clade containing *Ardeadactylus* and *Cycnorhamphus*. “*Pterodactylus scolopaciceps*” is distinct from *Pterodactylus antiquus,* located in a monophyletic clade that we name Aurorazhdarchidae *fam. nov*. (urn:lsid:zoobank.org:act:F19628C7-8675-4148-AA54-A1C431543513) in replacement of the *nomen nudem* Protazhdarchidae Frey et al. 2011 [6], which does not comply with ICZN article 29.1. Aurorazhdarchidae *fam. nov*. contains “*P. scolopaciceps*” and its sister taxon *Gladocephaloideus* Lü, Ji, Wei and Liu, 2011 [45] (IG-CAGS-08-07) at its base, followed stepwise by *Cycnorhamphus* and the more derived sister taxa *Ardeadactylus longicolum* (Meyer, 1854 [7]) and *Aurorazhdarcho micronyx* (Meyer, 1856 [5]). Thus Aurorazhdarchidae *fam. nov*. is defined as *Aurorazhdarcho*, “*Pterodactylus scolopaciceps”*, their most recent common ancestor and all of its descendants. The Aurorazhdarchidae *fam. nov.* is based on six synapomorphies: (Ch. 13) the dorsal and ventral margins of the rostrum are mostly sub-parallel; (Ch. 42) the quadratojugal is robust, broadly separating the quadrate from the jugal (Fig. 13b & c); (Ch. 61) teeth are graded large to small caudally; (Ch. 68) the dentition is restricted to the prenarial rostrum; (Ch. 98) the pteroid is longer than half the length of the ulna (∼65% of the ulna in those with complete pteroids); and (Ch. 114) the prepubic plate (distal expansion) projects more posteroventrally than anterodorsally. In this cladogram Aurorazhdarchidae *fam. nov*. is the sister taxon to the Ornithocheiroidea + Azhdarchoidea, their most recent common ancestor and all of its descendants.

The topology warrants further discussion, however the cladistic analysis was not performed in order to test pterodactyloid relationships as a whole, but merely to test the monophyly of *Pterodactylus*. Therefore a more detailed review of pterosaur phylogeny and the results noted here is required (Vidovic in prep.).

## Discussion

Linear measurements of specimens assigned to *P. antiquus* [2] were compared in a series of graphical analyses, to demonstrate how well supported two morphotypes contained within the hypodigm are. In these graphical analyses there is consistently strong support for the relationship between specimens referred to morphotype two. The taxonomic relationship among specimens of morphotype one was not as well supported as morphotype two, but the morphotype one assemblage is found to be distinct from morphotype two. There were some instances of strong support for a relationship among all specimens, but equally, the results frequently demonstrated no relationship for all the specimens collectively. From these analyses it is clear that morphotype two is a valid species, referable to “*P. scolopaciceps*”. Both morphotypes one and two share affinities with *P. antiquus*, but neither display consistent relationships with this taxon, therefore “*P. scolopaciceps*” is unlikely to be congeneric with *Pterodactylus*. The distinguishing features of “*P. scolopaciceps*”, as demonstrated by these analyses, are the shape and length of the skull, the elongation of the cervical vertebrae, and a relatively small pes when compared to other pterodactyloids of a similar size. These features are not restricted to one morphological unit, and suggest that “*P. scolopaciceps*” occupied a different ecological niche to *P. antiquus* and the specimens studied as morphotype one. We suggest that the elongate metacarpal is consistent with wading, whilst the long neck and rostrum are conducive to low level feeding. Therefore, “*P. scolopaciceps*” was possibly a wading pterosaur, occupying a similar ecological niche to the modern Snipe, although we consider it likely that ecological niches changed through ontogeny.

Further examination of the taxonomic validity of “*P. scolopaciceps*” using cladistic methods confirms that it is not congeneric with *P. antiquus*. “*P. scolopaciceps*” and its sister taxon *Gladocephaloideus* are found at the base of a monophyletic clade also containing *Cycnorhamphus, Aurorazhdarcho* and *Ardeadactylus*, but not *P*. *antiquus*. “*P. scolopaciceps*” is distinguished from *Gladocephaloideus* and *Cycnorhamphus* by a more extensive dentition (skull length occupied by the dentition: *Gladocephaloideus* ∼20%; *Cycnorhamphus* ∼7%; “*P*. *scolopaciceps*” ∼45%) and a less depressed dorsal slope to the skull, which is more similar to that of *Ardeadactylus*. Indeed, ontogenetically variable features, such as the angle of the quadrate to the dental margin may be all that separates “*P*. *scolopaciceps*” from *Ardeadactylus* in our cladistic analysis. However, both *Ardeadactylus* and *Aurorazhdarcho* are distinct from “*P. scolopaciceps*” in the proportions of the wing metacarpal to other wing elements. In both *Ardeadactylus* and *Aurorazhdarcho* the wing metacarpal (MCIV) exceeds the ulna (ulna is 79% and 87% of MCIV length respectively), but is shorter than the first wing phalanx (WPh1) (MCIV is 83% and 79% of WPh1 length respectively). In “*P. scolopaciceps*” the wing metacarpal is shorter than the ulna and first wing phalanx (Fig. 2A), which is true of all size classes studied. Moreover the gradient of the regression line comparing these elements suggests that the wing metacarpal would never exceed the ulna in length. Furthermore specimens of “*P. scolopaciceps*” lack the small bony triangular headcrest present on *Ardeadactylus*, which is also referred to Aurorazhdarchidae *fam. nov*., although this could be ontogenetically variable. “*P.*” *scolopaciceps* also has a curved humeral shaft, and a narrow, spatulate prepubis that is similar to *Pterodactylus*, clearly distinguishing it from *Cycnorhamphus, Ardeadactylus* and *Aurorazhdarcho*. *Aurorazhdarcho* [6] is distinct from *Ardeadactylus* [2] in the extent of contact between the ischium and pubis, the sternal morphology and the gracility of its bones (ulna length/width quotient- *Ardeadactylus* = 14; *Aurorazhdarcho* = 21).

**Figure 14:**
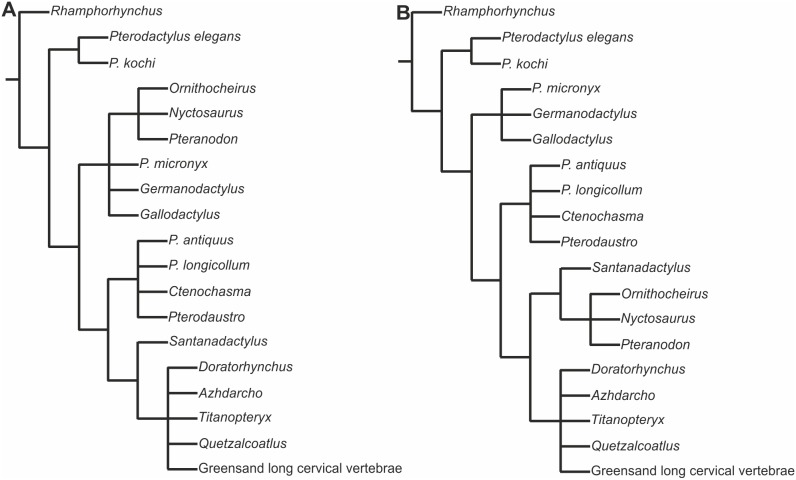
Cladograms based on the results of a cladistic analysis by Howse [43]. Pterodactylus elegans = Ctenochasma elegans; Pterodactylus micronyx = Aurorazhdarcho micronyx; Gallodactylus = Cycnorhamphus; Pterodactylus longicollum = Ardeadactylus longicollum; Doratorhynchus = Pterodactyloidea incertae sedis. (?azhdarchid?); Titanopteryx = Arambourgiana; Greensand long cervical vertebrae = no specimen number or reference given.

In the very first cladistic analysis of the Pterosauria, Howse [43], using only characters from cervical vertebrae, found a polytomic grouping of taxa that approximates the Azdarchidae of Unwin [46]. He also found *Pterodactylus kochi* to form a polytomy with *Pterodactylus elegans* ( = *Ctenochasma elegans*) at the base of Pterodactyloidea, whereas *P*. *antiquus* fell into a polytomy with *P*. *longicollum* ( = *Ardeadactylus*) in a more crownward position (Fig. 14). Since then, Unwin [33] combined *P*. *antiquus* and *P*. *kochi* into a single taxonomic unit, which fell into a polytomy with Lonchodectidae and Ctenochamatidae. Concurrently Kellner [26] analysed *P*. *antiquus* and *P*. *kochi* as separate taxonomic units, and found that together they formed a polytomy with *Germanodactylus*, noting that there is no synapomorphy to unite the two *Pterodactylus* species with each other relative to *Germanodactylus*. More recently, Wang et al. [41] found the same relationship as in Kellner [26]. However, Lü et al. [30] found that *Pterodactylus* formed a polytomy with *Cycnorhamphus* and Gnathosaurinae + Ctenochasmatinae in Ctenochasmatoidea. We suggest that the failure to resolve this polytomy may in part lie in the combining of two distinct taxa within *Pterodactylus*. In the cladistic analysis presented here we analysed the holotype specimens of *Pterodactylus antiquus* (BSP AS I 739); *P*. *kochi* (SMF R 404/BSP AS XIX) and “*P. scolopaciceps*” (BSP AS V 29 a/b). In addition we coded some characters for “*P*. *scolopaciceps*” from specimen BSP 1937 I 18 described by Broili [17] due to the poor preservation of the holotype. As a consequence of our analyses we place “*P. scolopaciceps*” in the new genus *Aerodactylus* gen. nov. urn:lsid:zoobank.org:act:D7B8AA15-6742-4BE3-B030-9CECA966086E.

## Systematic Palaeontology

PTEROSAURIA Kaup, 1834 [47]

MONOFENESTRATA Lü et al., 2010 [45]

PTERODACTYLOIDEA Plieninger, 1901 [31]

AURORAZHDARCHIDAE *fam. nov*.

*AERODACTYLUS* gen. nov.

Fig. 1 and Fig. 3F

### Type species

*Aerodactylus scolopaciceps* (Meyer, 1860 [8]).

### Content

The type species *Aerodactylus scolopaciceps* gen. nov. is the only species currently contained in *Aerodactylus.*

### Diagnosis

As for type and only species.

### Etymology

Aero = wind (Greek) + dactylus = finger (Greek), a common suffix in pterosaur names. The name derives from the Nintendo Pokémon Aerodactyl, a fantasy creature made up of a combination of different pterosaurian features. It seemed a pertinent name for a genus which has been synonymous with *Pterodatylus* for so long due to a combination of features.

### Synonymy

1850 *Pterodactylus longirostris Meyer*, p. 199 [48]

*When Meyer originally described the specimen he referred it to what is now known as* Pterodactylus antiquus

1860 *Pterodactylus scolopaciceps* Meyer, p. 33 [8]

1883 *Pterodactylus kochi* (H. v. Meyer) Zittel p. 25 [14]

1901 *Pterodactylus scolopaciceps* von Meyer; Seeley p. 105 [49]

1938 *Pterodactylus scolopaciceps* H. v. Meyer; Broili p. 146 [17]

1970 *Pterodactylus scolopaciceps* H. v. Meyer, 1850; Wellnhofer p.22 [15]

1970 *Pterodactylus kochi* Wagner, 1837; Wellnhofer p. 22 [15]

### Holotype

BSP AS V 29 a/b: part and counterpart, with a complete skeleton of a small juvenile.

### Referred material

BSP 1883 XVI 1 (Fig. 2Bi, ii and 3D) and counterpart MCZ 1505: a complete skeleton of a large individual. BSP 1975 I 221: a complete skeleton on a slab, in left lateral view. BSP 1937 I 18 (Fig. 2A and 3E): a complete specimen with soft tissue, in dorsal and right lateral view. Example of Frey & Martill [20]: a complete specimen with soft tissue in right lateral view. NHMW 1975/1756: part and counterpart, a complete skeleton with soft tissue.

### Locality and horizon

Solnhofen Limestone, Malm Zeta 2, Solnhofen, Bavaria, Germany.

### Diagnosis

(None of the following are autapomorphies, but together form a unique combination of characters) A pterodactyloid pterosaur with elongate cervical vertebrae (approximately three times longer than they are wide at the mid-centrum corpus). The rostrum is very elongate (RI = ∼7), terminating in a point, making the upper and lower jaws superficially like the beak of a Eurasian Woodcock. The jaws contain 16 teeth per-jaw, per-side, which are more sparsely distributed caudally (tooth spacing is equal to adjacent tooth width mesially, and up to four times adjacent tooth width distally) and terminate rostral to the nasoantorbital fenestra. Dorsal surface of skull concave, subtly depressed rostral to the cranial table. Orbit low and elongate (more than one and a half times longer than it is deep). Wing-metacarpal elongate, but shorter than both ulna and first wing-phalanx. The pteroid is approximately 65% of the total length of the ulna, straight and extremely thin (length is greater than ten times its width, and it is less than one third the width of the ulna). The humeral shaft is slightly bowed, curving cranially, just anconal of the midsection. The plate of the prepubis gradually sweeps away from the scapus, the lateral extremity of the plate is angular (∼90°), and the distal edge is sub-rounded (as in *P. antiquus*).

### Taxonomic Remarks

Although PTH 1962.148 demonstrates similar proportions and morphologies to *Aerodactylus* gen. nov. it possesses a straight humeral shaft and a bowed pteroid. It is possible that this example is a juvenile of *Ardeadactylus*. This suggestion is supported by the presence of a very small bony crest dorsal to the frontals and caudal to the parietals, perhaps an anchoring point for a more extensive soft tissue crest. The separation of the pubis and ischium suggests a possible affinity with *Ardeadactylus* (personal observations), although it is more likely due to early ontogeny. The wing metacarpal of PTH 1962.148 is short relative to its ulna, which is not consistent with *Ardeadactylus*. The “*P. micronyx*” specimens assigned to *Aurorazhdarcho* have long wing metacarpals, suggesting that the proportions of the ulna and wing metacarpal do not change significantly through ontogeny.

## Anatomical Description

### Cranial skeleton

The cranial anatomy of *Aerodactylus scolopaciceps* gen. nov. is typified by an elongate orbit and skull, with a subtle depression in the dorsal surface of the rostrum. The premaxilla is fused to the maxilla even in the smallest juvenile (BSP AS V 29a/b), making it difficult to tell where the maxilla starts. Dorsal to the nasoantorbital fenestra the premaxilla extends caudally to the caudal third of the orbit. The distal tip of the premaxilla has a convex profile, caudal of the first two teeth the rostrum is sub-parallel. The four most mesial teeth are tentatively identified as the premaxillary dentition, which consists of simple conical teeth. In the premaxilla of BSP 1937 I 18 the caudal two teeth exhibit a tumescence in the cervical region of the crown, however this may be a taphonomic feature caused by the expansion of infilling minerals in the pulp cavity. The teeth in the premaxilla and the mesial maxillary teeth are perpendicular to the jaw margin, but the shorter crowns of the distal most teeth are slightly procumbent, whereas all the teeth in the dentary are slightly procumbent. The majority of the dentition is nearly isodont, but the four distal teeth are up to two and a half times smaller than the larger teeth. In the mesial part of the jaw, the distance between teeth is approximately equal to the adjacent tooth width, but distally the gaps become much greater, up to four times the width of the adjacent tooth. Tooth spacing is approximately equal to the adjacent tooth width in the mesial part of the jaw, but distally the gaps become much greater. The dentition terminates immediately rostral to the nasoantorbital fenestra vacuity. The vacuity of the nasoantorbital fenestra is significantly less than a third of the total length of the premaxilla. Rostral to the nasoantorbital fenestra a thin sheet of maxillary bone occupies the space between the caudal process of the premaxilla and caudal, dentulous portion of the caudal process of the maxilla. The maxilla extends half the length of the nasoantorbital fenestra vacuity, contacting the ventral margin of the splint-like jugal maxillary process. The ventral margin of the maxilla is slightly concave with the distal procumbent teeth projecting into the void, but the ventral margin of the maxilla and premaxilla appears straight. The jugal is very elongate and slender, with the dorsal lacrimal process (DLPJ) displaced rostral, relative to the ventral apex. Compared to the rest of the jugal, the DLPJ is very broad at its base (approximately twice the breadth of the maxillary and caudal processes). The ventral apex of the jugal curves ventrally, to form a smooth sweeping curve with the quadratojugal, terminating at the articular process of the quadrate. The caudal process of the jugal extends to the caudal margin of the infratemporal fenestra, where it contacts the postorbital and possibly a postfrontal, which is unclear and poorly defined in all specimens. The quadratojugal is a short rectangular bone, located at the anteroventral margin of the infratemporal fenestra. Adjacent to the quadratojugal, the quadrate is slender and elongate, extending caudally to the occipital region of the skull, caudal to the orbit. The long axis of the quadrate lies at approximately 170 degrees to the dental plane of the upper jaw. The infratemporal fenestra is bounded on its ventral margin by the quadrate. The infratemporal fenestra lies entirely beneath the orbit, with a poorly defined mass of bone comprising the postorbital and squamosal behind it. The supratemporal fenestra is located caudal to the infratemporal fenestra in the ventral portion of the skull. The squamosals, parietals and frontals form a smooth, rounded cranium, meeting the premaxilla and nasals above the orbit. The nasals underlie the premaxilla and extend approximately half way into the nasoantorbital fenestra vacuity. The descending process of the nasal descends steeply and is long, but does not reach the maxilla. In BSP 1937 I 18 elements of the palate have been displaced into the nasoantorbital fenestra and orbit, tentatively identified here as the pterygoids and ectopterygoids. The lacrimal meets the dorsal process of the jugal, separating the orbit and nasoantorbital fenestra. The dorsal half of the lacrimal is as broad as the ventral part of the DLPJ, but there is a distinctive step making it considerably thinner ventrally (a quarter of the width of the dorsal portion). Due to the step in the lacrimal the orbit is low and long, with a length just over one and a half times its depth.

The lower jaw is similar in morphology to the anterior portion of the rostrum, becoming gradually more robust caudally (three and a half times deeper than the anterior part). The mandible is approximately straight along almost its entire length, only curving ventrally immediately rostral to the retroarticular process. The retroarticular process is short, broad (equal length and depth), wedge-like and in line with the sweep of the jaw. There is no intramandibular ridge of the splenial preserved in the specimens studied, but it is suggested to have been present to some extent considering its extensive presence in both *Ardeadactylus* and *Cycnorhamphus* [50] which share a close common ancestor to *Aerodactylus* gen. nov.

### Postcranial skeleton

#### Axial skeleton

The axial skeleton is typified by a long cervical series, consisting of elongate cervical vertebrae (cervical vertebra five, three times the length of its width), followed by a comparitively short PCRW and a short tail (for a pterodactylioid). There are 7–8 cervical vertebrae. The eighth ?cervical vertebra is half the length of the seventh and located between the scapulae, but it has a similar morphology to the other cervical vertebrae. However, the first dorsal rib is not clearly preserved, leaving some ambiguity over the identification of an eighth cervical vertebra. The cervical vertebrae are typically procoelous and lack cervical ribs. The neural spines of the cervical vertebrae are very low and long (at least ten times longer than they are tall), and the neural spine of the axis is indistinct. The cervical centra are constricted slightly caudal to the craniocaudal centre when seen in dorsal view, from this point the pre-exapophyses and postexapophyses project cranio/caudolaterally from the centrum corpus. The pre-exapophyses are more elongate than the postexapophyses. The dorsal vertebrae are 1.5–2 times longer than wide. The neural spines are taller than they are long and perpendicular to the centrum. The specimens lack a notarium, and lack fusion between vertebral centra, although this could be due to the juvenile condition of the specimens studied. The thoracic ribs are poorly preserved and it is difficult to distinguish the articular ends proximal to the vertebrae. There are four sacral vertebrae with transverse processes fused to the iliac blade. Caudal of the sacral vertebrae there are 15 small caudal vertebrae which together are approximately two thirds the length of the femur, and ∼15% of the total vertebral column length.

#### Appendicular skeleton

**Figure 15:**
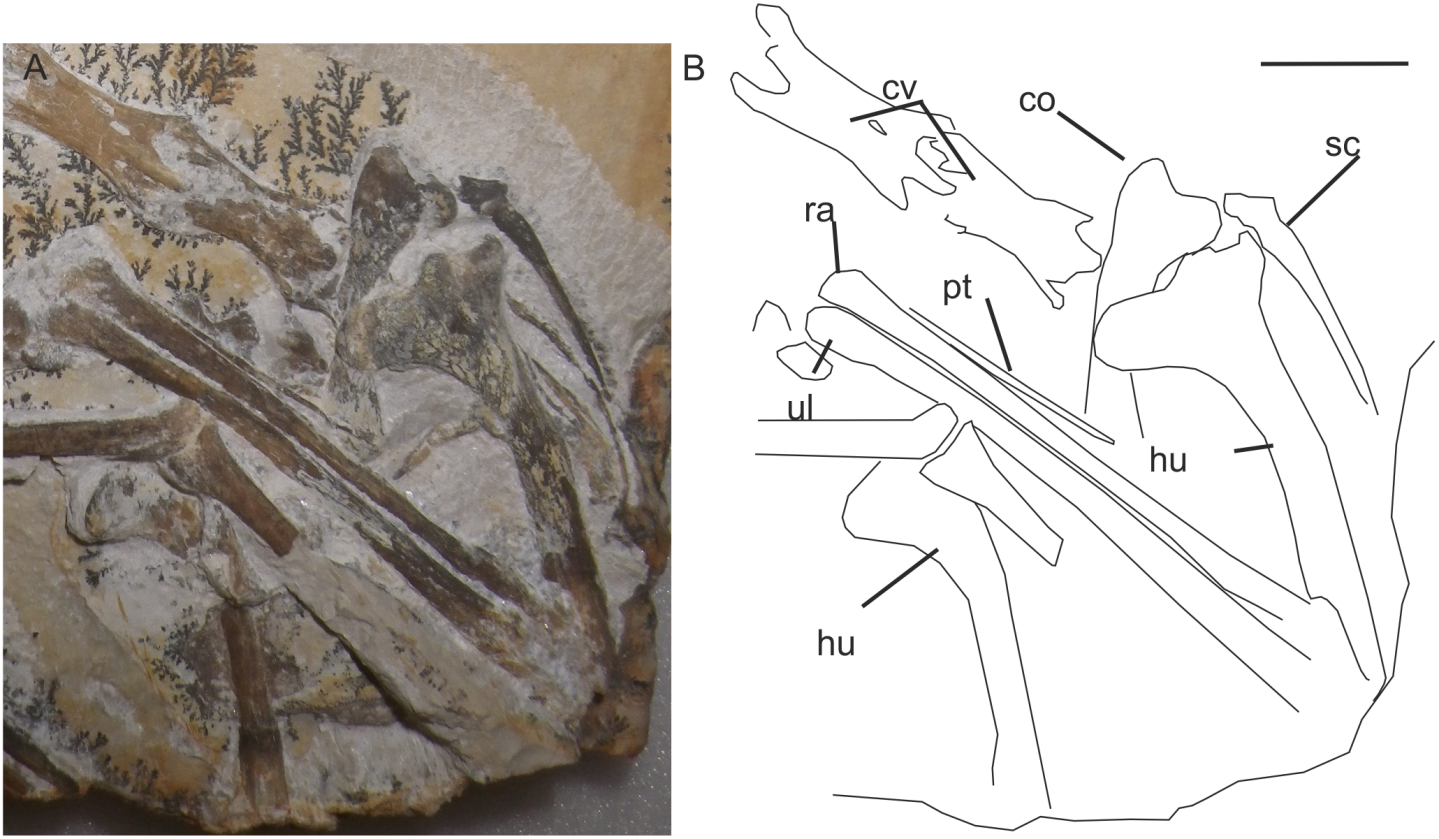
A private specimen of *Aerodactylus* demonstrating the shoulder girdle morphology. A) A photograph of a private specimen, which plots onto all graphs on the regression line belonging to morphotype two, thus is identified as *Aerodactylus scolopaciceps* gen. nov. The area of the photograph illustrates the morphology of the coracoid and humerus. The humerus is more similar to that of *Aurorazhdarcho*, but the proportions of the wing metacarpal are similar to *Aerodactylus scolopaciceps* gen. nov. B) Interpretive line diagram: co, coracoid; cv, cervical vertebra; hu, humerus; pt, pteroid; ra, radius; sc, scapula; ul, ulna; wph1, wing-phalanx one; wph2, wing-phalanx two. Scale bar = 10 mm.

The sternum is not well preserved in any specimens that can be confidently assigned to *Aerodactylus* gen. nov. (PTH 1962.148 has a similar sternum morphology to *P.antiquus*). The scapula and coracoid are unfused, possibly due to the specimens studied not being fully mature. The scapula extends caudally, and is elongate, spatulate with a deflection proximal to the glenoid fossa. The coracoid is very broad proximal to the glenoid (Fig. 15), as in *Cycnorhamphus, Aurorazhdarcho* and *Ardeadactylus*. The humeral shaft is slightly bowed cranially (it is straight in *Cycnorhamphus*, *Ardeadactylus* and *Aurorazhdarcho*), the deltopectoral crest continuously sweeps from the shaft towards the humeral head. The deltopectoral crest is approximately equal in width to the humeral head, and is rounded at its cranial end. The caudal margin of the humerus is somewhat sigmoidal along its length due to a slightly flared humeral head proximally and an exaggerated flexure of the distal condyles in the opposite direction. The medial and lateral epicondyles of the humerus are approximately equal in size.

The ulna and radius are almost equal in width, and approximately three times the length of the humerus. There are no detectable muscle scars, proximal tubercle of the radius, or olecranon process of the ulna. The pteroid is approximately 65% of the total length of the ulna and extremely thin, lacking the proximal curvature seen in *Pterodactylus antiquus*. The pteroid length is an autapomorphy of Aurorazhdarchidae *fam. nov.* with *Aerodactylus* gen. nov., *Cycnorhamphus* and *Aurorazhdarcho* sharing a straight pteroid, compared to the sigmoidal pteroid of *Ardeadactylus.* The wing metacarpal is elongate, but does not exceed the length of the ulna, and the growth allometry of the sampled specimens suggests that adult specimens would maintain this condition. The first wing phalanx is ∼20% longer than the wing metacarpal but fractionally (1–2%) shorter than the ulna. The second wing phalanx is approximately the same length, or slightly (∼1%) shorter than the first, the third is 85% of the total length of wing phalanx one, and the fourth is 70% the total length of wing phalanx one. The first three wing phalanges are straight, while the fourth phalanx has a slight caudal curvature. The manus compares favourably with that of *Pterodatylus*, but not with *Aurorazhdarcho*.

The pelvis is poorly preserved in the majority of specimens. The preacetabular process is a straight, elongate semicircle. The postacetabular process is a short, posterodorsally directed process. It is not possible to tell if the pubis is completely fused to the ischium as in *Aurorazhdarcho*, or if it is a largely distinct process as in *Ardeadactylus*. The prepubis consists of an elongate scapus, smoothly sweeping out to a broadly symmetrical plate. The prepubic plate is angular at its lateral margin and rounded on its distal margin.

The femur is slightly bowed caudally and is approximately 65% of the total tibia length. The tibia is long and straight. The fibula has not been detected. It is also hard to distinguish the tarsals, due mainly to crushing and calcite crystal mineralization on the distal end of the tibia. The middle phalanges of pedal digit four are square, and the proximal phalanx exceeds the length of the distal phalanx. The pedal unguals are significantly (40%) smaller than those of the manus. The fifth pedal digit consists of a single small phalanx, less than half the size of the robust fifth metatarsal.

#### Soft tissue

Soft tissues are preserved in several pterosaur specimens from the Solnhofen Limestone Formation, and have been detected in both Pterodactyloidea and Rhamphorhynchidae [12], [13], [14], [15], [16]. These include wing membranes, integument and integumentary structures and soft tissue head crests (Fig. 2). For a detailed description of the soft tissue of *Aerodactylus scolopaciceps* gen. nov. refer to Wellnhofer [16] and Frey et al. [13], [20]. All pterosaurs from the Solnhofen Limestone with a soft tissue cone-like head crest can be referred to *Aerodactylus* gen. nov.

The preserved integument of BSP 1937 I 18 demonstrates a gular pouch, propatagium, tenopatagium, brachiopatagium and uropatagium. The gular pouch extends from the mandible, directly below the descending process of the nasal, to the caudal end of the fourth cervical vertebra. Close to the cranial end of the sixth cervical vertebra the integument sweeps towards the shoulders and continues to the pteroid to form the propatagium. The wing membrane is broad close to the body, but is narrow along the spar of the wing finger. The uropatagium is attached approximately a third of the way up the tibia, proximal to the ankle, and broadly sweeps to the hip, resulting in two independent membranes [15], [17], [51]. There is also evidence for pycnofibres on the neck of *Aerodactylus* gen. nov.[20], as in *Gladocephaloideus* [45].

## Conclusions

The taxonomy of *Pterodactylus* is complex due to a suite of plesiomorphic characters that are retained in early ontogeny. Recently, Bennett [2] referred “*Pterodactylus longicollum*” to the new genus; *Ardeadactylus,* and synonymised *P. kochi* with the type and (in our opinion) only species *P. antiquus.* This synonymy is likely to be erroneous considering that the focussed statistical analyses and a cladistics analysis presented here have demonstrated that “*P. kochi”* includes at least one other taxon. One such taxon within ‘*P. kochi*’ is “*Pterodactylus scolopaciceps*” Meyer 1860 [8]. “*Pterodactylus scolopaciceps*” is demonstrated to differ from *Pterodactylus antiquus* in the morphology of its skull and pteroid. Our statistical analysis finds that the two differ in the proportions of the orbit, humerus and pes, and so *Aerodactylus* gen. nov. is erected for the reception of “*P. scolopaciceps*”. Remaining specimens of *P. kochi* are found to have only a few proportions in common with *P. antiquus.* Notably, the cervical vertebrae of *P. kochi* are much shorter (nearly half the length when scaled to the same breadth) than those of *P. antiquus*, and thus we reject Jouve’s [24] and Bennett’s [2] synonymy. Further examination of the remaining specimens contained within *P. kochi* is required but our analysis suggests that it is generically distinct, and thus validates *Diopecephalus* Seeley, 1871 [52] (Vidovic and Martill in prep.). Furthermore “*Germanodactylus rhamphastinus*” is distinct from *G. cristatus* and requires further taxonomic evaluation. It is probable that “*G. rhamphastinus*” also belongs in *Diopecephalus*, as it has a straight dorsal rostrum, teeth beneath the nasoantorbital fenestra and short cervical vertebrae (Vidovic and Martill in prep.).

## Supporting Information

File S1 **Cladistic procedures.** An explanation of the cladistics procedure, with a character list, a table of continuous data, a data matrix and results.
(DOCX)

File S2 **Statistical procedures.** Three pages of statistical tests on various datasets.
(XLSX)
