# Correction: *Pterodactylus scolopaciceps* Meyer, 1860 (Pterosauria, Pterodactyloidea) from the Upper Jurassic of Bavaria, Germany: The Problem of Cryptic Pterosaur Taxa in Early Ontogeny

**DOI:** 10.1371/journal.pone.0118015

**Published:** 2015-02-06

**Authors:** The PLOS ONE Staff

There is an error in reference 25. The correct reference is: Andres B, Ji Q (2008) A new pterosaur from the Liaoning province of China, the phylogeny of the Pterodactyloidea, and convergence in their cervical vertebrae. Palaeontology 51: 453–469.
