# Supplementary material for: Pterodactylus scolopaciceps Meyer, 1860 (Pterosauria, Pterodactyloidea) from the Upper Jurassic of Bavaria, Germany: The Problem of Cryptic Pterosaur Taxa in Early Ontogeny
Source: PLoS One. 2014 Oct 22;9(10):e110646. doi: 10.1371/journal.pone.0110646 (PMC4206445; doi:10.1371/journal.pone.0110646)
Supplement: File S1 — Cladistic procedures. An explanation of the cladistics procedure, with a character list, a table of continuous data, a data matrix and results. (DOCX) [file pone.0110646.s001.docx]

**S1: Supporting Information**

*Pterodactylus* *scolopaciceps* Meyer, 1860 (Pterosauria, Pterodactyloidea) from the Upper Jurassic of Bavaria, Germany: the problem of cryptic taxa in early ontogeny

*by* STEVEN U. VIDOVIC, and DAVID M. MARTILL

Steven U. Vidovic [*Steven.Vidovic@port.ac.uk*]; David M. Martill [*David.Martill@port.ac.uk*], Palaeobiology Research Group, School of Earth and Environmental Sciences, University of Portsmouth, Burnaby Building, Burnaby Road, Portsmouth, PO1 3QL, United Kingdom

**Table of contents**

**S1.1.1.** Character generation and coding methods

**S1.1.2.** Continuous character list

**S1.1.3.** Discrete character list

**S1.2.1.** Table of measurements and continuous characters

**S1.2.2.** Data matrix

**S1.2.3.** TNT procedure

**S1.3.1.** TNT results

**S1.3.2.** Cladograms

**S1.4.1.** Images of “*P. antiquus*” specimens

**S1.1.1.**

The 127 characters are a compilation of new and previously used characters, comprising 52 characters from Wang et al. (2009), Lü et al. (2010), references therein, and 75 new characters (see S.1.3). Ten of the new characters are continuous characters, 9 of which are ratios taken from direct measurements, and the remaining character was derived from the tooth count per half jaw. Some of the remaining 65 new characters are the result of atomising and modifying compound characters. Compound characters were split into their component characters so that the resulting tree topologies would not be affected by character construction (Brazeau, 2011). For example characters 18 to 23 code the information that Wang et al. (2009) (characters: 15, 16, 24 & 25) and Lü et al. (2010) (characters: 9 & 28) wrote into compound characters. The character states were coded from direct observations and information made available in the literature.

All continuous characters were scaled from 0-1, with 1 being equal to the maximum of the data range studied. The motivation for rescaling continuous characters was “to diminish the influence of measures of parts of very different sizes” as in Pereyra and Mound (2009). Additionally, quotient values with converse relationships between the elements examined were rescaled using the equation i=tan^-1^a/b and are marked with a ‘*’.

Taxa with multiple states for the same character are coded with an ‘&’ between the states. When there is uncertainty as to which state is applicable, but there is evidence for both ‘/’ is used between the states. Taxa with unknown states for a character are coded with ‘?’, those with non-applicable states are coded with ‘-’, all other character states are coded between 0 and 3.

**S1.1.2.**

Ch.1 Rostral index * (In reference to the rostral length over depth ratio as in Lü et al. 2010: Ch. 6) (tan^-1^[rostrum length/rostrum depth]).

Ch. 2 Preorbital rostrum length to prenarial rostrum length (preorbital length/rostrum length).

Ch. 3 PCRW to length of the neck (praecaudale Rumpfwirbelsäule)* (tan^-1^[PCRW length/neck length]).

Ch. 4 Length of the humerus to the PCRW (PCRW length/humerus length).

Ch. 5 Length of the ulna to the wing-metacarpal* (tan^-1^[ulna length/wing-metacarpal length]).

Ch. 6 Length of wing-phalanx 1 relative to wing-phalanx 2* (tan^-1^[wph1/wph2]).

Ch. 7 Length of wing-phalanx 3 relative to wing-phalanx 4 (wph3/wph4).

Ch. 8 Length of femur to PCRW (PCRW length/femur length).

Ch. 9 Length of tibia to femur (tibia length/femur length).

Ch. 10 Tooth count per jaw, per side.

**S1.1.3.**

Ch. 11 Tip of rostrum: downturned (0); upturned (1); in line with dental plane (2) (modified from Lü et al. 2010: Ch. 1 and Wang et al. 2009: Ch. 4).

Ch. 12 Tip of rostrum laterally expanded: absent (0); present (1) (Lü et al. 2010: Ch. 2 and Wang et al. 2009: Ch. 2).

Ch. 13 The dorsal margin of rostrum and the occlusal surface of the rostrum: are approximately parallel (0); immediately diverge (1) anterior to the NAOF.

Ch. 14 Dorsal margin of the rostrum anterior to the NAOF: straight (0); convex (1); concave (2).

Ch. 15 The dorsal margin of the skull above the NAOF: dorsally expanded (0); depressed (1); straight (2).

Ch. 16 Foramen on nasal process: present (0); absent (1) (After Wang et al. 2009: Ch. 21).

Ch. 17 Caudal periphery of the nasoantorbital fenestra is reclined (0); descending at approximately 90 degrees (1); reflexed (2).

Ch. 18 Headcrest: present (0); absent (1).

Ch. 19 If present, the headcrest: is isolated to the prenarial rostrum (0); extends over the NAOF (1); is isolated posterior of the NAOF (-).

*If Ch. 20 is coded with a 1 or ‘-’ Ch. 19 should be coded with a ‘-’.*

Ch. 20 If present, the headcrest is made up of: premaxilla (0); frontoparietals (1); premaxilla and frontoparietals (2).

*If Ch. 18 is coded with a 1 Ch. 20 should be coded with a ‘-’.*

Ch. 21 If present, the premaxillary headcrest is: low (length to depth ratio >1.5:1) (0); tall (length to depth ratio <1.5:1) (1).

*If Ch. 18 is coded with a 1, or Ch. 20 is coded with a 1 or 2 Ch. 21 should be coded with a ‘-’.*

Ch. 22 If present, the headcrest is made up of: smooth bone (0); fibrous bone (1).

*If Ch. 18 is coded with a 1 Ch. 22 should be coded with a ‘-’.*

Ch. 23 If present, fronto-parietal crest: flange-like (1); rod-like (2); sail-like (3).

*If Ch. 18 is coded with a 1, or Ch. 20 is coded with a 0 Ch. 23 should be coded with a ‘-’.*

Ch. 24 Soft tissue cone headcrest: present (0); absent (1).

*If Ch. 18 is coded with a 1 Ch. 20 should be coded with a ‘-’.*

Ch. 25 Premaxilla extends: to the orbit but not beyond its anterior margin (0); beyond the anterior margin of the orbit (1).

Ch. 26 Over the nasoantorital fenestra the premaxilla: tapers out (0); is parallel sided (1); expands (2).

Ch. 27 Ventral margin of the skull: sinusoidal (0); approximately straight (1); upward curving (2); downward curving (3) (modified from Lü et al. 2010: Ch. 11).

Ch. 28 Posterior margin of nasoantorbital fenestra: straight (0); concave (1) (modified from Lü et al. 2010: Ch. 22).

Ch. 29 Lacrimal process of the jugal: thin (0); broad (1) (modified from Wang et al. 2009: Ch. 23).

Ch. 30 Lacrimal process of the jugal: inclined anteriorly (0); perpendicular to the dental plane (1); reclined posteriorly (2).

Ch. 31 Orbit shape: subcircular, jugal processes at right angle (0); tall, oval, jugal processes at acute angle (1) (Lü et al. 2010: Ch. 23).

Ch. 32 Orbit: shorter than antorbital opening (0); slightly taller or equal (1) (Lü et al. 2010: Ch.24).

Ch. 33 Dorsal margin of orbit: level with dorsal margin of nasoantorbital opening (0); or below it (1) (Lü et al. 2010: Ch. 26).

Ch. 34 Suborbital opening: present (0); absent (1) (Wang et al. 2009: Ch. 14).

Ch. 35 The anterior-most extent of the inferior temporal fenestra is: under the anterior third of the orbit (0); under the middle third of the orbit (1); under the posterior third of the orbit (2).

Ch. 36 The posterior-most extent of the inferior temporal fenestra is: under the orbit (0); posterior of the orbit (1).

Ch. 37 Anterior margin of the inferior temporal fenestra: reclined posteriorly(0); nearly perpendicular to the occlusal plane (1).

Ch. 38 Frontal extends anterior to the lacrimal-jugal bar: absent (0); present (1) (Lü et al. 2010: Ch. 27).

Ch. 39 Distal ends of paroccipital processes: unexpanded (0); rounded, tongue-like flange (1) (Lu et al Ch. 33).

Ch. 40 Basispterygoid: short (0); elongated (three or more times the length of their width) (1) (Wang et al. 2009: Ch. 33).

Ch. 41 Quadrate: reclined posteriorly (0); subhorizontal (1) (after Lü et al. 2010: Ch. 34).

Ch. 42 The quadratojugal is robust (wider than quadrate) and the quadrate is distinct from the jugal: present (0); absent (1).

Ch. 43 Position of the jaw joint: under the posterior third of the orbit (0); under the middle third of the orbit (1); under the anterior third of the orbit (2) (Lü et al. 2010: Ch. 36).

Ch. 44 Medial palatal ridge of the premaxilla and maxilla: absent (0); present (1) (Lü et al. 2010: Ch. 37).

Ch. 45 The posterior palate is descended forming a “suspensorium”: present (0); absent (1).

Ch. 46 Mandibular rami: level with symphysis (0); elevated well above level of symphysis (1) (Lü et al. 2010: Ch. 45).

Ch. 47 Dentary bony sagittal crest: absent (0); present (1) (Lü et al. 2010: Ch. 46).

Ch. 48 The dorsal and ventral margins of the lower jaw, under the antorbital fenestra: bowed, diverging (0); equidistant/parallel (1); posteriorly convergent (2); posteriorly divergent (3).

Ch. 49 Jaw rami fused: distally (0); approximately halfway along (1); proximally (2).

Ch. 50 Splenial intramandibular shelf: present (0); absent (1).

Ch. 51 Splenial intramandibular shelf: present, medially-fused (0); present, medially-unfused (1).

*If Ch. 50 is coded with a 1 Ch. 51 should be coded with a ‘-’.*

Ch. 52 Lower jaw: extends beyond the rostrum (0); is in line with the rostrum (1); doesn't extend to the end of the rostrum (2).

Ch. 53 Retroarticular process: in line with jaw (0); ascending (1); descending (2).

Ch. 54 43.Dentition: present (0); absent (1) (Lü et al. 2010: Ch. 55).

Ch. 55 Teeth: in sockets (0); in a groove (1).

*If Ch. 54 is coded with a 1 Ch. 55 should be coded with a ‘-’.*

Ch. 56 Raised alveolar edges: present (0); absent (1).

*If Ch. 54 is coded with a 1 Ch. 56 should be coded with a ‘-’.*

Ch. 57 Teeth are: isodont (0); homodont/heterodont (1).

*If Ch. 54 is coded with a 1 Ch. 57 should be coded with a ‘-’.*

Ch. 58 Number of tooth morphologies: homodont (0); 2 (1); 3 (2).

*If Ch. 54 is coded with a 1, or Ch. 57 is coded with a 0 Ch. 58 should be coded with a ‘-’.*

Ch. 59 If heterodont, the tooth morphology in the lower jaw is: similar to that of the rostrum (0); different to that of the rostrum (1).

*If Ch. 54 is coded with a 1, or Ch. 57 is coded with a 0, or Ch. 58 is coded with a 0 Ch. 59 should be coded with a ‘-’.*

Ch. 60 Teeth: Regularly spaced (0); Irregularly spaced, closer spacing distally (1); irregularly spaced, closer spacing mesially (2).

*If Ch. 54 is coded with a 1 Ch. 60 should be coded with a ‘-’.*

Ch. 61 Tooth size distribution (crown height or alveolus width): graded; large to small caudally (0); graded; small to large caudally (1); grading up, then back down (2); ungraded, varying in size (festooned) (3).

*If Ch. 54 is coded with a 1, or Ch. 57 is coded with a 0 Ch. 61 should be coded with a ‘-’.*

Ch. 62 The distance between teeth is: equal to the adjacent tooth width (0); greater than the adjacent tooth width (1); less than the adjacent tooth width (2).

*If Ch. 54 is coded with a 1 Ch. 62 should be coded with a ‘-’.*

Ch. 63 The dentition is typified by: strongly recurved teeth (0); straight teeth (1); sinusoidal teeth (2).

*If Ch. 54 is coded with a 1 Ch. 62 should be coded with a ‘-’.*

Ch. 64 The dentition is typified by teeth that are: triangular (0) leaf shaped (1); parallel sided (2).

*If Ch. 54 is coded with a 1 Ch. 62 should be coded with a ‘-’.*

Ch. 65 Dentition typified by teeth with a: round cross-section (0); compressed cross-section (1).

*If Ch. 54 is coded with a 1 Ch. 62 should be coded with a ‘-’.*

Ch. 66 Teeth are typically: short and broad (approximately as tall as they are wide, or shorter) (0); tall (1); elongate (>5 times their width) (2).

*If Ch. 54 is coded with a 1 Ch. 62 should be coded with a ‘-’.*

Ch. 67 Dentition: extends to jaw tips (0); jaw tips toothless, but followed by tooth row (1) (Lü et al. 2010: Ch.59).

*If Ch. 54 is coded with a 1 Ch. 62 should be coded with a ‘-’*

Ch. 68 Extent of the dentition: not beneath the nasoantorbital fenestra (0); beneath the nasoantorbital fenestra (1).

*If Ch. 54 is coded with a 1 Ch. 62 should be coded with a ‘-’*

Ch. 69 Atlas and axis: unfused (0); fused (1) (Wang et al. 2009: Ch. 53).

Ch. 70 Postexapophyses on cervical vertebrae: absent (0); present (1) (Lü et al. 2010: Ch. 63 and Wang et al. 54).

Ch. 71 Lateral pneumatic foramen on centrum of the cervical: absent (0); present (1) (Lü et al. 2010: Ch. 64 and Wang et al. 2009: Ch. 55).

Ch. 72 Mid-series cervicals: short (approximately twice its minimum width or less) (0); elongate (approximately three times its minimum width or more) (1); very elongate (approximately 4 times its minimum width or more) (2) (modified from Lü et al. 2010: Ch. 65 and Wang et al. 2009: Ch. 56).

Ch. 73 Neural arch of cervicals: high (0); depressed down onto, or even confluent with, the centrum (1) (Lü et al. 2010: Ch. 67).

Ch. 74 Neural spines of mid-series cervicals extremely low or absent: present (0); absent (1) (modified from Lü et al. 2010: Ch. 68 and Wang et al. 2009: Ch. 58).

Ch. 75 Neural spines of mid-series cervicals: blade-like (0); spike-like (1) (modified from Lü et al. 2010: Ch. 68 and Wang et al. 2009: Ch. 58).

*If Ch. 74 is coded with a 0 Ch. 75 should be coded with a ‘-’.*

Ch. 76 Fusion of the dorsal vertebrae: present (0); absent (1) (modified from Lü et al. 2010: Ch. 69).

Ch. 77 Tail: longer than the femur (0); shorter than the femur (1).

Ch. 78 Filiform extensions of zygapophyses and hypapophyses: absent (0); present (1) (Lü et al. 2010: Ch. 72).

Ch. 79 Sternum: rectangular (0); triangular (1); semicircular (2) (modified from Lü et al. 2010: Ch. 73).

Ch. 80 Sternum has: perforated lateral margin (0); smooth lateral margin (1).

Ch. 81 Keel of sternum: equal in length to half the width of the plate (0); longer than half the width of the plate (1); shorter than half the width of the plate (2).

Ch. 82 Coracoid: less than two thirds length of scapula (0); from at least two thirds up to similar length to scapula (1); longer than scapula (2) (Lü et al. 2010: Ch. 75).

Ch. 83 Coracoid with well-developed brachial flange: absent (0); present (1) (Lü et al. 2010: Ch. 76).

Ch. 84 Coracoidal contact surface with sternum: articulation surface flattened, lacking posterior expansion (0); articulation surface oval, with posterior expansion (1) (Lü et al. 2010: Ch. 77).

Ch. 85 The cross-section of the scapula is: rounded (0); spatulate (1).

Ch. 86 73.Deltopectoral crest curving ventrally: present (0); absent (1) (modified from Wang et al. 2009: Ch. 73).

Ch. 87 Deltopectoral crest: proximally placed (0); or descended (1) (modified from Wang et al. 2009: Ch. 73).

Ch. 88 Deltopectoral crest: pinched at the shaft (0); even sided, or converging margins cranially (1).

Ch. 89 Cranial margin of the deltopectoral crest: squared off (0); rounded (1); nearly a point (2).

Ch. 90 Deltopectoral crest: warped (0); not warped (1).

Ch. 91 Medial (=ulnar) crest of the humerus: absent or reduced (0); present (1) (modified from Wang et al. 2009: Ch. 74).

Ch. 92 Pneumatic opening in palmar surface of humerus: absent (0); present (1) (Lü et al. 2010: Ch. 82 and Wang et al. 2009: Ch. 71).

Ch. 93 Pneumatic opening in anconal surface of humerus: absent (0); present (1) (Lü et al. 2010: Ch. 83 and Wang et al. 2009: Ch. 72).

Ch. 94 Distal end of humerus: D-shaped (0); triangular (1) (Lü et al. 2010: Ch. 85 and Wang et al. 2009: Ch. 75).

Ch. 95 Medial epicondyle of the humerus larger than the lateral epicondyle (0); or approximately equal in size (1).

Ch. 96 Diameter of radius: more than half the ulna (0); less (1) (Lü et al. 2010: Ch. 86).

Ch. 97 The articulation ends of the ulna are: much wider than the shaft (0); not much wider than the shaft (1).

Ch. 98 Pteroid: shorter than half the length of the ulna (0); longer that half the length of the ulna (1) (modified from Wang et al. 2009: Ch. 94).

Ch. 99 Pteroid bone (measured at the mid-shaft): at least approximately half the width of the radius (0); thinner than half the width of the radius (1); extremely thin, less than one quater the width of the radius (2).

Ch. 100 Pteroid bone is: straight (0); proximally bent, distally straight (1); bowed (2).

Ch. 101 Lateral carpal: is robust (0); is slender, much like the pteroid (1).

Ch. 102 Distal syncarpals: unfused (0); fused in a rectangular unit (1); fused in a triangular unit (2) (Wang et al. 2009: Ch. 78).

Ch. 103 (Functional) manus: present (0); absent (1).

Ch. 104 Unguals of manus and pes (measured from the middle of the articulatory end to the distal tip): similar in size (0); manual unguals twice the size, or more, of pedal unguals (1) (Lü et al. 2010: Ch. 95).

Ch. 105 Longitudinal ventral ridge on wing-phalanges two and three: absent (0); present (1) (Lü et al. 2010: Ch. 99).

Ch. 106 Wing phalanx IV: present (0); absent (1).

Ch. 107 Anterior profile, in lateral view, of pubis: convex or straight (0); slightly concave (1); deeply concave (2) (Lü et al. 2010: Ch. 104).

Ch. 108 Obturator foramen of pelvis: present (0); absent (1).

Ch. 109 (If present) the obturator foramen is: anterior to the acetabulum (0); medial to the acetabulum (1); posterior to the acetabulum (2).

*If Ch. 108 is coded with a 1 Ch. 109 should be coded with a ‘-’*

Ch. 110 Pre-acetabular process: straight (0); curving dorsally (1).

Ch. 111 Pubis: is as long or longer than the pre-acetabular process (0); is more than half the length of the pre-acetabular process (1); is less than half the length of the preacetabular process (2).

Ch. 112 Posterior margin of the ischium: angular (0); rounded (1).

Ch. 113 The post-acetabular-ischial notch is: approximately equal in depth to the acetabulum (0); larger than the acetabulum (1).

Ch. 114 Prepubic plate projects: more anterodorsally than posteroventrally (0); more posteroventrally than anterodorsally (1); equally in all directions (2).

Ch. 115 Prepubic plate is: rounded/rocker shaped (0); angular, square (1); angular, triangular (2); spatulate (3).

Ch. 116 Pre-pubis: spade-like (0); bifurcating (1).

Ch. 117 Prepubis is: equal in width to the ischium (at their widest points) (0); wider than the ischium (1); not as wide as the ischium (2).

Ch. 118 Femur caput: directed inward at about 135° (0); directed steeply almost parallel to long axis of femur shaft (1) (Lü et al. 2010: Ch. 108).

Ch. 119 Strongly bowed femur: absent (0); present (1) (Lü et al. 2010: Ch. 109).

Ch. 120 Prominent cranially directed tubercle on dorsal apex of external trochanter of femur: absent (0); present (1) (Lü et al. 2010: Ch. 110).

Ch. 121 Distal end of femur with: simple condylar morphology (0), complex condylar morphology (1) (modified from Lü et al. 2010: Ch. 112).

Ch. 122 Fibula reduced to a small splint or lost altogether: absent (0); present (1) (modified from Lü et al. 2010: Ch. 113).

Ch. 123 Fifth pedal digit: two phalanges (0); one very short phalange, or less (1) (Lü et al. 2010: Ch. 116).

Ch. 124 Terminal phalanx of pes digit V: approximately equal in size to preceding phalanx or metatarsal (0); shorter than preceding phalanx or metatarsal (1).

Ch. 125 Terminal phalanx of pedal digit V: straight (0); boomerang-shaped (1) (modified from Lü et al. 2010: Ch. 117 and Wang et al. 2009: Ch. 89).

Ch. 126 The phalanges of pedal digit IV are: proximal phalanx is larger than all those succeeding it (0) distal and proximal phalanxes are longer than those between them (2).

Ch. 127 Pedal digit IV phalanx II & III are: longer than they are wide (0); as wide, or wider than they are long (1).

**S1.2.1.**

| Taxa | Prenarial rostrum length | Prenarial rostrum depth | Ch. 1 | Preorbital length | Ch. 2 | Neck length | PCRW length | Ch. 3 | Humerus length | Ch. 4 | Ulna length | Wing-metacarpal length | Ch. 5 | Wing-phalanx1 length | Wing-phalanx 2 length | Ch. 6 | Wing-phalanx 3 length | Wing-phalanx 4 length | Ch. 7 | Femur length | Ch. 8 | Tibia length | Ch. 9 | Tooth number | Ch. 10 |
| --- | --- | --- | --- | --- | --- | --- | --- | --- | --- | --- | --- | --- | --- | --- | --- | --- | --- | --- | --- | --- | --- | --- | --- | --- | --- |
| *Anhanguera piscator* | 294 | 55 | 0.921 | 497 | 0.415 |  |  | ? |  | ? |  |  | ? |  |  | ? |  |  | ? |  | ? |  | ? | 23 | 0.153 |
| *Ardeadactylus longicollum* | 119 | 17 | 0.949 | 175 | 0.361 | 213 | 136 | 0.570 | 78 | 0.744 | 104 | 132 | 0.552 | 158 | 107 | 0.833 | 77 | 64 | 0.584 | 97 | 0.619 | 147 | 0.937 | 16 | 0.107 |
| *Aurorazhdarcho* |  |  | ? |  | ? |  | 130 | ? | 65 | 0.853 | 85 | 96 | 0.600 | 120 | 78 | 0.850 | 57 | 51 | 0.542 | 78 | 0.735 | 120 | 0.951 | ? | ? |
| *Caupedactylus* | 173 | 156 | 0.556 | 404 | 0.573 |  |  | ? | 175 | ? |  |  | ? |  |  | ? |  |  | ? |  | ? |  | ? | 0 | 0.000 |
| *Ctenochasma elegans* | 56 | 4 | 0.996 | 79 | 0.346 | 74 | 72 | 0.774 | 39 | 0.787 | 51 | 51 | 0.650 | 63 | 57 | 0.714 |  |  | ? | 34 | 0.934 | 55 | 1.000 | 55-60 | 0.367-0.400 |
| *Cycnorhamphus suevicus* | 61 | 12 | 0.915 | 106 | 0.426 | 108 |  | ? | 66 | ? | 88 | 109 | 0.562 | 133 | 117 | 0.726 | 86 | 70 | 0.596 | 76 | ? | 120 | 0.976 | 3-4 | 0.02-0.267 |
| *Darwinopterus* | 65 | 17 | 0.874 | 154 | 0.581 | 78 | 91 | 0.865 | 48 | 0.809 | 73 | 28 | 0.997 | 54 | 60 | 0.626 | 65 | 60 | 0.526 | 47 | 0.854 | 56 | 0.737 | 15 | 0.100 |
| *Darwinopterus linglongtaensis* | 40 | 10 | 0.881 | 89 | 0.546 |  |  | ? | 38 | ? | 58 | 22 | 1.000 | 44 | 50 | 0.617 | 52 | 52 | 0.485 | 38 | ? | 48 | 0.781 | ? | ? |
| *Dsungaripterus weii* | 205 | 52 | 0.879 | 380 | 0.455 |  |  | ? |  | ? |  |  | ? | 383 | 278 | 0.806 |  |  | ? |  | ? |  | ? | 12 | 0.080 |
| *Germanodactylus cristatus* | 47 | 11 | 0.891 | 92 | 0.480 | 84 |  | ? | 55 | ? | 73 | 63 | 0.711 | 80 | 81 | 0.666 | 68 | 56 | 0.589 | 55 | ? | 84 | 0.944 | 14-16 | 0.093-0.107 |
| *Germanodactylus rhamphastinus* | 76 | 21 | 0.865 | 156 | 0.503 | 136 | 104 | 0.655 | 58 | 0.765 | 92 | 70 | 0.762 | 119 | 81 | 0.831 | 71 |  | ? | 64 | 0.717 | 98 | 0.947 | 16 | 0.107 |
| *Gladeocephaloideus jingangshanensis* | 117 | 13 | 0.970 | 149 | 0.312 |  |  | ? |  | ? |  |  | ? |  |  | ? |  |  | ? |  | ? | 87 | ? | 13 | 0.087 |
| *Gnathosaurus subulidens* |  |  | ? |  | ? |  |  | ? |  | ? |  |  | ? |  |  | ? |  |  | ? |  | ? |  | ? | 30 | 0.200 |
| *Istiodactylus latidens* | 80 | 27 | 0.827 | 324 | 0.993 |  |  | ? |  | ? | 304 |  | ? |  |  | ? |  |  | ? | 189 | ? |  | ? | 12 | 0.080 |
| *Istiodactylus sinensis* | 79 | 26 | 0.832 | 265 | 0.823 |  |  | ? | 134 | ? | 241 | 163 | 0.808 | 268 | 239 | 0.720 | 198 |  | ? |  | ? |  | ? | 12 | 0.080 |
| *Ludodactylus* | 243 | 56 | 0.893 | 393 | 0.397 |  |  | ? |  | ? |  |  | ? |  |  | ? |  |  | ? |  | ? |  | ? | 23 | 0.153 |
| *Nyctosaurus gracilis* | 142 | 27 | 0.919 | 182 | 0.314 | 112 | 120 | 0.823 | 87 | 0.588 | 131 | 244 | 0.408 | 283 | 195 | 0.826 | 88 |  | ? | 77 | 0.688 | 89 | 0.715 | 0 | 0.000 |
| *Ornithocheirus mesembrinus* | 281 | 42 | 0.945 | 536 | 0.468 |  |  | ? |  | ? |  |  | ? |  |  | ? |  |  | ? |  | ? |  | ? | 17 | 0.113 |
| *Prejanopterus* |  |  | ? |  | ? |  |  | ? |  | ? |  |  | ? |  |  | ? |  |  | ? |  | ? |  | ? | ? | ? |
| *Pteranodon longiceps* | 504 | 61 | 0.964 | 648 | 0.315 |  |  | ? |  | ? | 84 | 164 | 0.392 |  |  | ? |  |  | ? | 24 | ? | 32 | 0.824 | 0 | 0.000 |
| *Pterodactylus antiquus* | 48 | 9 | 0.921 | 81 | 0.414 | 89 | 61 | 0.603 | 32 | 0.813 | 48 | 34 | 0.790 | 49 | 45 | 0.707 | 39 | 29 | 0.653 | 31 | 0.868 | 47 | 0.937 | 20-25 | 0.133-0.167 |
| *Pterodactylus kochi* |  | 6 | ? |  | ? | 44 | 68 | 1.000 | 29 | 1.000 | 43 | 30 | 0.796 | 40 | 36 | 0.716 | 32 | 25 | 0.621 | 30 | 1.000 | 42 | 0.865 | ? | ? |
| *Pterodactylus scolopaciceps* | 38 | 5 | 0.957 | 59 | 0.381 | 65 | 55 | 0.705 | 27 | 0.869 | 38 | 26 | 0.803 | 36 | 36 | 0.671 | 31 | 26 | 0.579 | 28 | 0.867 | 38 | 0.839 | 16 | 0.107 |
| *Pterodaustro guinazui* | 167 | 11 | 1.000 | 198 | 0.291 |  | 96 | ? | 65 | 0.630 | 102 | 73 | 0.786 | 110 | 111 | 0.667 | 84 | 68 | 0.599 | 59 | 0.718 | 93 | 0.974 | 100-150 | 0.667-1.000 |
| *Shenzhoupterus* | 96 | 30 | 0.842 | 185 | 0.473 | 132 | 105 | 0.674 | 83 | 0.540 | 93 | 155 | 0.447 | 144 | 100 | 0.823 | 65 | 36 | 0.876 | 100 | 0.463 | 139 | 0.859 | 0 | 0.000 |
| *Sinopterus dongi* | 41 | 27 | 0.657 | 104 | 0.622 | 93 | 111 | 0.876 | 60 | 0.789 | 88 | 93 | 0.627 | 111 | 95 | 0.737 | 68 | 33 | 1.000 | 69 | 0.710 | 99 | 0.887 | 0 | 0.000 |
| *Tapejara navigans* | 91 | 164 | 0.337 | 259 | 0.698 |  |  | ? |  | ? |  |  | ? |  |  | ? |  |  | ? |  | ? |  | ? | 0 | 0.000 |
| *Tapejara wellnhoferi* | 48 | 62 | 0.438 |  | ? |  |  | ? | 69 | ? | 95 | 105 | 0.609 | 156 | 66 | 1.000 |  |  | ? | 72 | ? | 94 | 0.807 | 0 | 0.000 |
| *Tupandactylus imperator* | 77 | 217 | 0.227 | 314 | 1.000 |  |  | ? |  | ? |  |  | ? |  |  | ? |  |  | ? |  | ? |  | ? | 0 | 0.000 |
| *Tupuxuara* | 285 | 105 | 0.809 | 729 | 0.627 |  |  | ? |  | ? |  |  | ? |  |  | ? |  |  | ? |  | ? |  | ? | 0 | 0.000 |
| *Zhejiangopterus* | 124 | 28 | 0.896 | 238 | 0.471 |  |  | ? |  | ? |  |  | ? |  |  | ? |  |  | ? |  | ? |  | ? | 0 | 0.000 |
| *Zhenyuanopterus* | 351 | 43 | 0.963 | 471 | 0.329 | 285 | 255 | 0.732 | 207 | 0.525 | 267 | 230 | 0.712 | 353 | 284 | 0.763 | 212 | 140 | 0.735 | 212 | 0.531 | 199 | 0.580 | 42 | 0.280 |

**S1.2.2.**

&[cont]

*Anhanguera piscator*  0.921 0.415 ? ? ? ? ? ? ? 0.153

*Ardeadactylus longicollum* 0.949 0.361 0.570 0.744 0.552 0.833 0.584 0.619 0.937 0.107

*Aurorazhdarcho micronyx* ? ? ? 0.853 0.600 0.850 0.542 0.735 0.951 ?

*Azhdarcho lancicollis* ? ? ? ? ? ? ? ? ? ?

*Caupedactylus deliradamus* 0.556 0.573 ? ? ? ? ? ? ? 0.000

*Ctenochasma elegans* 0.996 0.346 0.774 0.787 0.650 0.714 ? 0.934 1.000 0.367-0.400

*Cycnorhamphus suevicus* 0.915 0.426 ? ? 0.562 0.726 0.596 ? 0.976 0.02-0.267

*Darwinopterus modularis* 0.874 0.581 0.865 0.809 0.997 0.626 0.526 0.854 0.737 0.100

*Darwinopterus linglongtaensis* 0.881 0.546 ? ? 1.000 0.617 0.485 ? 0.781 ?

*Dsungaripterus weii*  0.879 0.455 ? ? ? 0.806 ? ? ? 0.080

*Germanodactylus cristatus* 0.891 0.480 ? 0.000 0.711 0.666 0.589 ? 0.944 0.093-0.107

*G. rhamphastinus* 0.865 0.503 0.655 0.765 0.762 0.831 ? 0.717 0.947 0.107

*Gladocephaloideus*  0.970 0.312 ? ? ? ? ? ? ? 0.087

*Gnathosaurus subulidens* ? ? ? ? ? ? ? ? ? 0.200

*Istiodactylus latidens*  0.827 0.993 ? ? ? ? ? ? ? 0.080

*Istiodactylus sinensis* 0.832 0.823 ? ? 0.808 0.720 ? ? ? 0.080

*Ludodactylus sibbicki*  0.893 0.397 ? ? ? ? ? ? ? 0.153

*Nyctosaurus gracilis* 0.919 0.314 0.823 0.588 0.408 0.826 ? 0.688 0.715 0.000

*Ornithocheirus mesembrinus* 0.945 0.468 ? ? ? ? ? ? ? 0.113

*Prejanopterus curvirostra* ? ? ? ? ? ? ? ? ? ?

*Pteranodon longiceps 0.964 0.315 ? ? 0.392 ? ? ? 0.824 0.000*

*Pterodactylus antiquus* 0.921 0.414 0.603 0.813 0.790 0.707 0.653 0.868 0.937 0.133-0.167

*Pterodactylus kochi* ? ? 1.000 1.000 0.796 0.716 0.621 1.000 0.865 ?

*Pterodactylus scolopaciceps* 0.957 0.381 0.705 0.869 0.803 0.671 0.579 0.867 0.839 0.107

*Pterodaustro guinazui* 1.000 0.291 ? 0.630 0.786 0.667 0.599 0.718 0.974 0.667-1.000

*Shenzhoupterus chaoyangensis* 0.842 0.473 0.674 0.540 0.447 0.823 0.876 0.463 0.859 0.000

*Sinopterus dongi* 0.657 0.622 0.876 0.789 0.627 0.737 1.000 0.710 0.887 0.000

*Tapejara navigans* 0.337 0.698 ? ? ? ? ? ? ? 0.000

*Tapejara wellnhoferi* 0.438 ? ? ? 0.609 1.000 ? ? 0.807 0.000

*Tupandactylus imperator* 0.227 1.000 ? ? ? ? ? ? ? 0.000

*Tupuxuara* 0.809 0.627 ? ? ? ? ? ? ? 0.000

*Zhejiangopterus linhaiensis* 0.896 0.471 ? ? ? ? ? ? ? 0.000

*Zhenyuanopterus* 0.963 0.329 0.732 0.525 0.712 0.763 0.735 0.531 0.580 0.280

&[num]

*Anhanguera piscator* 1 1 [0/1] [1&2] [1&2] 0 [1/2] 0 0 0 0 0 - 1 1 0 2 1 1 1 1 1 0 1 1 0 0 1 0 1 0 1 2 0 1 ? 1 1 0 1 - 1 0 0 0 1 1 2 0 2 3 1 [0&1] 0 [0&1] 1 0 1 ? 1 1 0 0 1 1 ? 1 0 2 0 1 1 0 0 0 1 0 1 1 0 0 0 1 1 1 0 0 ? ? ? 1 2 0 1 0 0 2 0 1 ? ? 0 1 ? ? ? ? 1 1 0 0 1 1 - 0 ? ?

*Ardeadactylus longicollum* [0&2] 0 0 2 1 ? 2 0 - 1 - 0 1 1 1 0 1 [0&1] 1 0 0 1 0 1 1 0 0 0 0 ? [0/1] 0 1 1 ? 1 0 ? 0 0 0 1 ? 0 0 1 1 0 - 2 0 [0&2] 0 0 0 1 0 0 0 1 0 2 1 1 0 0 1 0 [0/2] 1 ? 1 1 0 1 1 1 1 1 1 0 ? 0 0 1 0 1 1 [1/2] 2 0 1 0 ? 0 0 0 1 - 0 0 0 1 1 1 0 0 0 0 0 0 1 ? ? ? ? ?

*Aurorazhdarcho micronyx* ? ? ? ? ? ? ? ? ? ? ? ? ? ? ? ? ? ? ? ? ? ? ? ? ? ? ? ? ? ? ? ? ? ? ? ? ? ? ? ? ? ? ? ? ? ? ? ? ? ? ? ? ? ? ? ? ? ? ? ? ? ? ? ? ? 1 1 0 2 1 2 1 1 1 1 1 1 1 1 1 0 1 0 0 1 1 1 1 1 2 ? 1 0 0 0 0 0 1 - ? 2 0 ? 2 0 0 1 1 0 1 0 1 1 1 0 0 1

*Azhdarcho lancicollis* [1/2] 0 1 2 ? ? ? ? ? ? - ? ? ? ? ? ? ? ? ? ? ? ? ? ? ? ? ? ? ? ? ? ? ? ? ? ? ? ? ? ? ? ? 1 - - - - - - - - - - - - - - 1 0 0 2 1 0 - 0 ? ? ? ? ? ? 1 ? 0 0 1 0 1 1 0 1 0 0 0 ? 1 ? ? ? ? 1 0 ? 1 0 ? ? ? ? ? ? ? ? ? ? ? 0 ? 1 0 ? ? ? ? ? ?

*Caupedactylus deliradamus* 0 0 1 2 0 ? 0 0 1 2 1 0 1 1 0 0 0 1 0 2 1 0 1 1 0 0 0 1 ? 1 0 1 2 0 0 0 0 2 0 1 - 1 ? 1 - - - - - - - - - - - - - - ? ? ? ? ? ? ? ? ? ? 0 0 2 1 0 1 1 0 0 1 1 1 0 1 1 0 1 ? ? ? ? ? ? ? ? ? ? ? ? ? ? ? ? ? ? ? ? ? ? ? ? ? ? ? ? ? ? ? ?

*Ctenochasma elegans* 1 0 0 2 2 1 1 1 - - - - - - 0 0 1 1 1 1 0 1 0 1 [0/1] 1 0 0 ? ? 1 ? 2 0 ? ? 0 3 2 0 0 1 2 0 0 1 1 0 - 0 0 0 1 2 0 2 0 1 0 ? 0 1 1 1 0 1 1 0 ? ? ? 1 ? ? 1 1 0 1 1 1 0 ? ? 0 1 0 1 0 0 1 0 ? 0 ? 0 0 2 ? ? 1 2 0 ? 0 1 0 [0/2] 0 1 0 1 0 1 1 0 ? ?

*Cycnorhamphus suevicus* [0&1] 0 0 2 2 1 1 0 - 1 - 0 1 1 1 0 0 1 1 1 1 1 0 1 0 1 0 0 0 ? 0 0 2 0 ? ? 0 ? 0 0 1 1 0 0 0 1 0 - - - - ? 1 2 0 1 0 0 1 1 0 0 1 1 1 1 ? ? 2 1 2 1 1 0 1 1 0 1 1 1 0 ? 0 0 1 0 1 1 2 0 0 1 0 ? 0 0 2 0 0 1 1 0 1 1 1 0 2 0 0 0 0 1 ? ? ? ? ?

*Darwinopterus modularis* 2 0 1 0 [0&2] ? 1 0 1 0 0 1 - 1 1 0 1 1 1 1 1 1 0 1 1 1 0 0 0 1 0 1 1 0 ? 0 0 1 0 1 - 1 [0/2] 0 0 1 1 0 - 2 2 1 1 0 0 1 0 1 ? 0 0 0 1 1 0 1 0 1 2 1 2 0 0 0 1 1 1 1 1 1 0 0 0 0 1 0 1 0 1 2 0 0 0 0 0 0 0 1 - 0 0 0 0 1 0 0 0 0 0 1 1 ? 0 1 1 1 0

*Darwinopterus linglongtaensis* 2 0 1 2 [1&2] 0 1 0 1 0 0 1 - 1 1 1 1 0 0 1 0 1 0 1 2 1 0 1 0 ? 0 1 0 ? ? ? 0 [1/2] 0 1 - 1 ? 0 0 1 0 - - 2 - [0&1] 1 0 0 1 0 0 ? 0 0 0 1 1 0 1 0 1 2 1 2 1 0 0 1 1 0 1 1 1 0 0 0 0 1 0 1 0 1 1 1 0 0 0 0 0 2 0 1 0 0 0 1 ? 0 0 1 0 0 0 ? 0 0 0 1 1 1

*Dsungaripterus weii* [1&2] 0 1 2 2 ? 1 0 1 2 0 1 2 1 1 1 0 0 1 [0&2] 0 0 0 0 2 1 1 0 1 1 0 1 2 1 ? 0 0 0 0 1 - 1 [0/2] 0 0 0 1 0 - 2 2 [1&2] 1 0 0 0 1 1 ? 1 1 0 1 0 - ? 1 0 ? ? ? ? ? ? ? ? ? ? ? ? ? ? 0 0 0 ? ? ? ? ? ? 1 ? ? 0 0 2 1 - ? ? ? ? ? ? ? ? 0 1 1 0 1 ? ? ? ? ?

*Germanodactylus cristatus* 2 0 1 0 2 1 1 0 1 2 0 1 1 1 1 0 [0/1] 0 0 1 1 1 0 1 1 0 0 0 1 ? 0 1 2 0 0 ? 0 3 0 1 - 1 2 0 0 1 1 0 - 2 2 [1&2] 1 0 1 0 1 1 0 1 ? 0 1 0 - 0 1 0 ? ? ? 1 1 1 1 1 0 0 1 1 0 ? ? 0 0 0 1 ? 0 1 ? ? 0 1 0 0 ? ? ? ? ? ? ? ? 2 0 ? 0 1 1 0 1 1 ? ? 0 1

*G. rhamphastinus* 2 0 1 0 2 1 [1/2] 0 1 0 0 1 - 1 1 0 1 ? 0 1 1 1 0 1 0 0 0 ? ? ? 0 1 2 0 ? ? 0 3 0 ? ? 1 2 0 0 1 1 0 - 2 2 1 1 0 0 1 0 1 0 1 0 0 1 1 0 1 1 0 2 1 2 1 0 0 1 1 0 1 1 1 0 1 0 0 1 0 1 0 1 0 0 1 0 0 0 0 0 0 0 0 1 0 1 2 0 0 0 0 1 0 0 0 1 1 0 ? ?

*Gladocephaloideus* 2 0 0 2 2 ? [0/1] 1 - - - - - - 0 1 [0/1] ? ? ? 0 1 0 1 1 0 0 0 0 ? 1 ? 2 ? ? ? 0 3 ? ? ? 1 2 0 0 1 1 0 - [0/2] 0 1 0 2 0 [1/2] 0 0 ? ? ? [1/2] ? ? ? ? ? ? ? ? ? ? ? ? ? ? ? ? ? ? ? ? ? ? ? ? ? ? ? ? ? ? ? ? ? ? ? ? ? ? ? ? ? ? ? ? ? ? ? ? ? 1 ? ? ? 0 1

*Gnathosaurus subulidens* 2 1 0 0 2 ? 1 0 1 0 0 1 - 1 1 0 1 1 1 1 0 1 0 1 0 1 0 0 0 1 1 ? 2 0 ? ? 0 3 [1/2] ? ? 1 ? 0 0 1 - 0 - 2 2 [1&2] 2 2 0 2 0 0 ? ? ? ? ? ? ? ? ? ? ? ? ? ? ? ? ? ? ? ? ? ? ? ? ? ? ? ? ? ? ? ? ? ? ? ? ? ? ? ? ? ? ? ? ? ? ? ? ? ? ? ? ? ? ? ? ? ? ?

*Istiodactylus latidens* 1 0 0 [1&2] 2 ? 0 1 - - - - - - 1 1 1 0 0 2 1 1 0 1 1 ? 0 0 ? ? 0 1 2 0 0 ? 0 ? 0 1 - 1 ? 0 0 1 0 - - 2 - [1&2] [0&1] 1 1 0 0 0 ? ? 1 ? ? ? ? 0 ? ? ? ? ? 2 0 1 0 1 ? 1 ? 0 ? ? 1 1 1 1 1 0 2 0 1 2 ? ? ? ? ? ? ? ? ? ? ? ? ? ? ? 1 0 1 0 ? ? ? ? ? ?

*Istiodactylus sinensis* 2 0 0 [1&2] 0 ? 0 1 - - - - - - 0 1 0 0 0 2 1 1 0 1 1 1 0 0 0 ? 0 1 2 0 ? ? 0 1 0 ? ? 1 0 0 0 1 0 - - 2 - [1&2] [0&1] 1 1 0 0 0 1 1 ? ? 0 1 1 ? ? ? ? ? ? 2 0 0 0 ? 1 1 0 0 ? ? ? ? 1 1 1 ? ? ? ? 2 0 ? 0 0 ? ? ? ? ? ? ? ? ? ? ? ? 0 ? 0 1 ? ? ? ? ?

*Ludodactylus sibbicki* 1 0 1 [1&2] 1 0 2 0 - 1 - 0 2 1 0 0 2 1 1 0 1 1 0 1 1 1 0 0 0 ? 0 1 2 0 1 1 0 1 0 1 - 1 0 0 0 1 1 2 0 2 3 [1&2] [0&1] 0 0 1 0 1 ? ? ? ? ? ? ? ? ? ? ? ? ? ? ? ? ? ? ? ? ? ? ? ? ? ? ? ? ? ? ? ? ? ? ? ? ? ? ? ? ? ? ? ? ? ? ? ? ? ? ? ? ? ? ? ? ? ? ?

*Nyctosaurus gracilis* 1 0 1 2 1 ? 1 0 - 1 - 0 3 1 0 0 2 1 1 1 0 1 0 1 1 1 0 0 0 0 0 1 0 0 1 0 0 1 2 1 - 0 2 1 - - - - - - - - - - - - - - 1 1 0 0 0 1 1 0 1 0 0 0 1 2 1 1 0 1 1 0 1 1 1 1 ? ? 1 1 1 1 0 1 1 2 1 - 0 1 2 0 1 1 1 0 1 2 3 1 0 1 1 1 1 1 ? ? ? ? ?

*Ornithocheirus mesembrinus* 2 [0/1] [0/1] [0&1] 2 ? [1/2] 0 0 2 1 0 2 1 1 1 1 1 1 0 1 1 0 1 1 1 0 0 0 0 0 1 2 0 0 1 1 0 0 1 - 1 1 0 0 1 1 [1/2] 0 2 3 [0&1] [0/1] 0 0 1 0 1 ? 1 1 0 0 1 1 0 ? ? 2 1 ? 1 0 0 0 1 0 1 ? 0 ? 0 1 1 1 ? ? ? ? ? ? ? ? ? ? ? 0 ? ? 1 ? ? ? ? ? ? ? ? ? ? ? ? ? ? ? ? ?

*Prejanopterus curvirostra* 1 0 0 2 ? ? ? 1 - - - - - - ? ? 2 ? ? ? ? ? ? ? ? ? ? ? ? ? ? ? ? 1 ? ? 0 ? 0 1 - ? ? 0 0 0 ? ? ? 1 1 1 1 [0/2] 0 [0/1] 0 ? ? ? ? ? ? ? ? ? ? ? ? ? ? ? 0 0 ? ? 0 ? ? 1 0 ? ? 0 0 ? ? ? ? ? ? ? ? ? 0 ? 2 1 - ? ? 0 1 2 1 0 2 0 1 1 ? ? ? ? ? ? ?

*Pteranodon longiceps* 1 0 1 2 1 ? [0&1] 0 - 1 - 0 2 1 1 0 2 1 1 2 1 1 0 1 0 1 0 1 0 1 0 1 2 0 1 0 0 2 2 1 - 2 0 1 - - - - - - - - - - - - - - 1 1 1 0 0 1 1 0 1 0 0 0 1 2 0 0 0 0 0 1 0 0 0 1 0 1 1 1 1 ? ? 1 ? 2 0 ? 0 0 [0/1] 0 2 0 1 0 1 2 3 1 0 1 1 1 1 1 1 ? 0 ? ?

*Pterodactylus antiquus* 2 0 [0&1] 0 2 0 1 1 - - - - - 1 1 1 1 1 1 1 0 1 0 1 1 1 0 0 0 ? 0 1 1 0 ? 0 0 1 0 1 - 1 2 0 0 1 1 0 - 2 0 [0&1] [0/1] 0 0 1 0 1 1 0 ? 1 1 1 0 1 1 0 1 1 0 1 0 0 1 1 0 1 1 1 0 ? ? ? 1 0 1 0 1 [1/2] 0 0 0 1 0 0 [0/1] ? ? 0 2 1 1 1 0 0 2 0 1 ? ? 0 ? ? ? 0 1

*Pterodactylus kochi* 2 0 1 0 2 ? 2 ? ? ? ? ? ? 1 1 1 1 1 1 1 0 1 0 1 1 1 0 0 0 ? 0 1 1 0 ? ? 0 1 0 1 - ? 0 0 0 1 1 0 - ? [0/2] 1 1 0 ? 0 ? 1 0 ? 0 0 1 1 0 1 1 0 ? ? ? ? ? ? 1 1 0 1 1 1 0 ? ? ? 1 0 1 0 1 2 ? 0 0 0 0 0 2 ? ? 1 ? 0 0 2 0 0 0 0 1 ? 1 ? 1 0 0 0 1

*Pterodactylus scolopaciceps* 2 0 0 2 1 1 1 0 - - - - - 0 1 1 1 1 1 1 0 1 0 1 1 [0/1] 0 ? 0 ? 1 0 2 0 ? 0 0 3 0 1 - 1 2 0 0 1 1 0 - 2 0 [1&2] 1 0 0 0 0 0 1 2 0 2 1 1 0 1 1 0 ? ? ? 0 0 ? 1 1 0 1 1 1 0 ? ? 0 ? 0 1 1 2 0 0 ? 0 0 0 0 2 ? ? 0 1 ? ? 1 0 0 ? 0 1 0 0 ? 1 1 0 0 1

*Pterodaustro guinazui* 1 0 0 2 2 1 2 1 - - - - - 1 0 1 2 1 1 1 0 1 0 1 1 1 0 0 ? ? 0 1 1 0 ? ? 0 3 ? ? ? 1 2 0 1 1 1 1 1 0 2 2 1 2 0 2 0 1 ? 1 1 1 1 1 [0&1] ? 1 0 ? ? ? ? ? 1 ? 1 0 1 1 1 0 ? ? 0 1 0 1 ? ? ? 0 1 0 0 0 0 ? ? ? 1 ? ? ? ? ? ? ? 0 1 1 ? 0 1 1 0 0 0

*Shenzhoupterus chaoyangensis* 0 0 1 [0&2] [0&1] ? 0 0 - 2 - 0 2 1 1 0 3 0 0 2 1 0 1 1 ? ? 0 0 1 ? 0 1 ? ? ? ? 0 2 1 1 - 1 0 1 - - - - - - - - - - - - - - ? ? ? 1 1 0 - ? 1 0 ? ? ? ? ? ? ? 0 0 1 1 1 ? ? ? ? ? 1 1 ? ? ? ? ? 0 ? 0 0 ? ? ? ? ? ? ? ? ? ? ? ? 1 ? 0 1 1 ? ? ? ?

*Sinopterus dongi* 0 0 1 2 0 ? 1 0 1 2 0 0 2 1 1 0 3 0 0 1 1 0 1 1 2 1 0 1 1 ? 0 1 2 ? ? ? 1 2 [0&1] 1 - 1 0 1 - - - - - - - - - - - - - - 1 ? ? ? ? 0 - 1 1 0 ? ? ? 1 0 1 1 0 0 1 1 1 ? ? ? ? ? 0 1 0 2 1 0 ? 0 0 0 0 ? ? ? ? ? ? ? ? ? ? ? ? 0 ? 1 1 ? ? ? ? ?

*Tapejara navigans* 0 0 1 2 0 ? 0 0 1 2 1 [0&1] 1 1 0 0 0 1 0 2 1 0 1 1 0 0 0 0 1 1 0 1 2 0 0 ? ? ? ? ? ? ? ? 1 - - - - - - - - - - - - - - ? ? ? ? ? ? ? ? ? ? ? ? ? ? ? ? ? ? ? ? ? ? ? ? ? ? ? ? ? ? ? ? ? 1 ? ? ? ? ? ? ? ? ? ? ? ? ? ? ? ? ? ? ? ? ? ? ? ? ?

*Tapejara wellnhoferi* 0 0 1 2 0 1 0 0 1 2 1 0 2 1 1 0 0 0 0 2 1 0 1 1 1 1 0 1 ? ? 0 1 2 0 0 0 1 2 0 1 - 1 0 1 - - - - - - - - - - - - - - 0 1 1 0 0 0 - ? ? ? 0 0 2 1 1 1 1 0 0 1 0 1 0 1 0 0 0 0 0 ? ? ? ? 1 0 0 0 0 1 0 ? 1 2 ? ? ? ? ? ? 0 1 1 1 1 1 ? 0 ? ?

*Tupandactylus imperator* 0 0 1 [1&2] 0 ? 0 0 1 2 1 [0&1] 2 1 1 0 0 1 0 2 1 0 1 1 1 1 0 1 ? ? 0 1 2 ? ? ? 1 2 1 1 - 1 0 1 - - - - - - - - - - - - - - ? ? ? ? ? ? ? ? ? ? ? ? ? ? ? ? ? ? ? ? ? ? ? ? ? ? ? ? ? ? ? ? ? ? ? ? ? ? ? ? ? ? ? ? ? ? ? ? ? ? ? ? ? ? ? ? ? ? ?

*Tupuxuara* 2 0 1 [0&2] [0&2] ? [0/1] 0 1 2 0 1 1 1 1 [0/2] 0 0 0 2 1 0 1 1 0 0 0 1 1 ? 0 1 2 0 0 ? 1 0 1 1 - 1 0 1 - - - - - - - - - - - - - - ? ? ? ? ? ? ? ? ? ? ? ? ? ? ? ? ? ? ? ? ? ? ? ? ? ? ? ? ? ? ? ? ? ? ? ? ? ? ? ? ? ? ? ? ? ? ? ? ? ? ? ? ? ? ? ? ? ? ?

*Zhejiangopterus linhaiensis* 2 0 1 0 [0&2] ? 1 1 - - - - - - 0 0 1 0 0 1 0 0 1 1 [1/2] 1 0 1 0 ? 0 1 1 ? ? ? 0 2 2 ? ? 1 0 1 - - - - - - - - - - - - - - ? 0 0 2 1 0 - 0 1 0 ? ? ? 1 1 ? 0 0 0 0 0 1 0 ? 0 0 ? 0 1 1 0 0 ? 1 0 0 1 0 0 ? ? ? ? ? ? ? ? ? ? 0 1 1 0 1 1 ? 0 ? ?

*Zhenyuanopterus* 2 0 0 2 [1/2] 1 1 0 - [1/2] ? 0 1 1 1 0 [0/1] 1 0 1 1 1 0 1 1 1 0 1 0 ? 0 1 2 ? ? ? 0 2 2 ? ? 1 [0/1] 0 0 1 1 0 0 1 [2/3] 1 [0&1] 2 [0&1] [1/2] 0 1 ? 1 ? 1 0 1 1 0 1 0 2 0 ? 1 1 0 0 ? 0 1 0 0 0 0 ? ? 1 0 1 0 0 1 1 2 0 1 0 0 ? ? ? ? ? ? ? ? ? ? ? 1 0 1 0 0 ? ? ? ? ?

;

**S1.2.3.**

The analysis was run in TNT 1.1 (Goloboff *et al.* 2008), last updated September 2013, on a PC with an AMD FX 4350 (4.2 GHz Quad core) CPU and 8 GB (RAM) memory. The RAM available to the program was set to the maximum 1GB.

*Darwinopterus modularis* was set as the outgroup. There were 33 active taxa and 127 characters. Ten of the characters used continuous states, consequently the data format was set to 32 states. All discrete states were given equal weights. In the first phase of tree searching a ‘new technology’ search was used to recover the shortest tree possible. To ensure the search was exhaustive, ratcheting was set to its maximum number of iterations. The settings are as follows:

Get trees from… Driven search (33 taxa at level 15) with initial additional sequences = 5

Find minimum length 1 time

Random seeds set to 16239 (using rseed* command)

Replace existing trees

Auto-constrain

Use… Sectorial search parameters:

RSS – Factor for number of selections = 43; Min. sector size = 18; Max. sector size = 18

CSS – Rounds = 3; Min. sector size = 10

For RSS and CSS globally swap every… 2 changes in sectors of size below 75; 10 changes in sectors of size above 75

For selections of size… above 75 use 6 drifting cycles; below 75 use 3 starts and fuse trees 0 times

Use… Ratchet parameters:

Stop perturbation phase when 20 substitutions made, or 99% swapping completed

Perturbation phase... 4 up-weighting probability, 4 down-weighting probability. Use alternate equal weights.

Number of iterations… 100,000 total number, 0 auto-constrained.

Use… Tree fusing parameters:

3 rounds, swapping after exchanging, start from best tree

When using driver… use fusing to multiply optimal trees

The second phase of tree searching used the ‘traditional search’ function to find the maximum number of MPTs. The search used trees saved to the RAM from the ‘new technology’ search as the starting trees. A TBR (Tree Bisection Reconnection) swapping algorithm was used to find the maximum topologies of the same length or shorter.

**S1.3.1.**

**Initial TNT new technology run**

Number of rearrangements tried = 11,567,908,651

2 trees saved

Tree length = 409.145

CI = 0.394

RI = 0.555

**TNT traditional search**

Start swapping from 2 tree, score 409.145 steps

Number of rearrangements tried = 22,846

2 tree saved

Tree length = 409.145

CI = 0.313

RI = 0.365

**S1.3.2.**


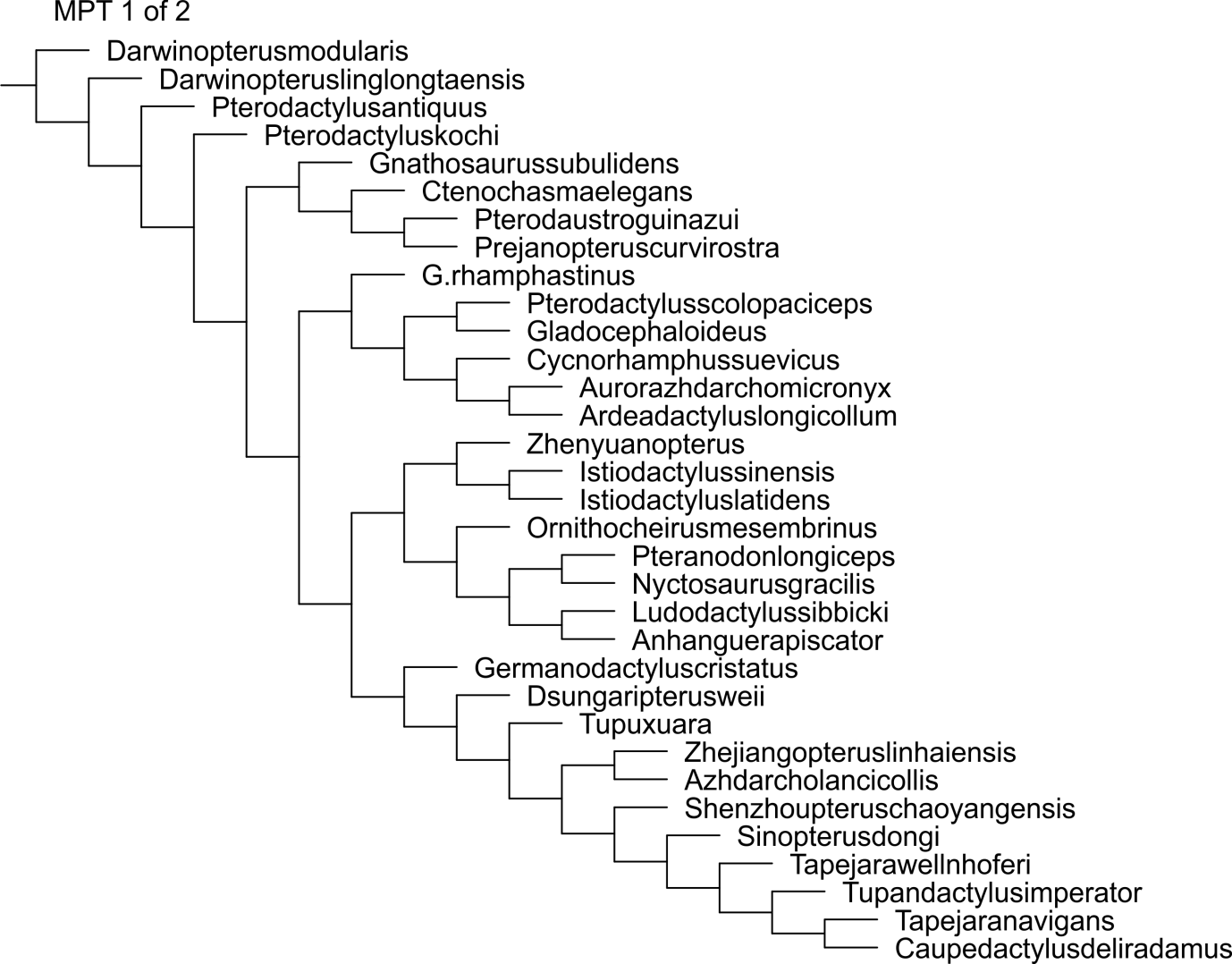


MPT 1 of 2 recovered. The same topology was resolved in both search functions (i.e. NT search and Traditional).


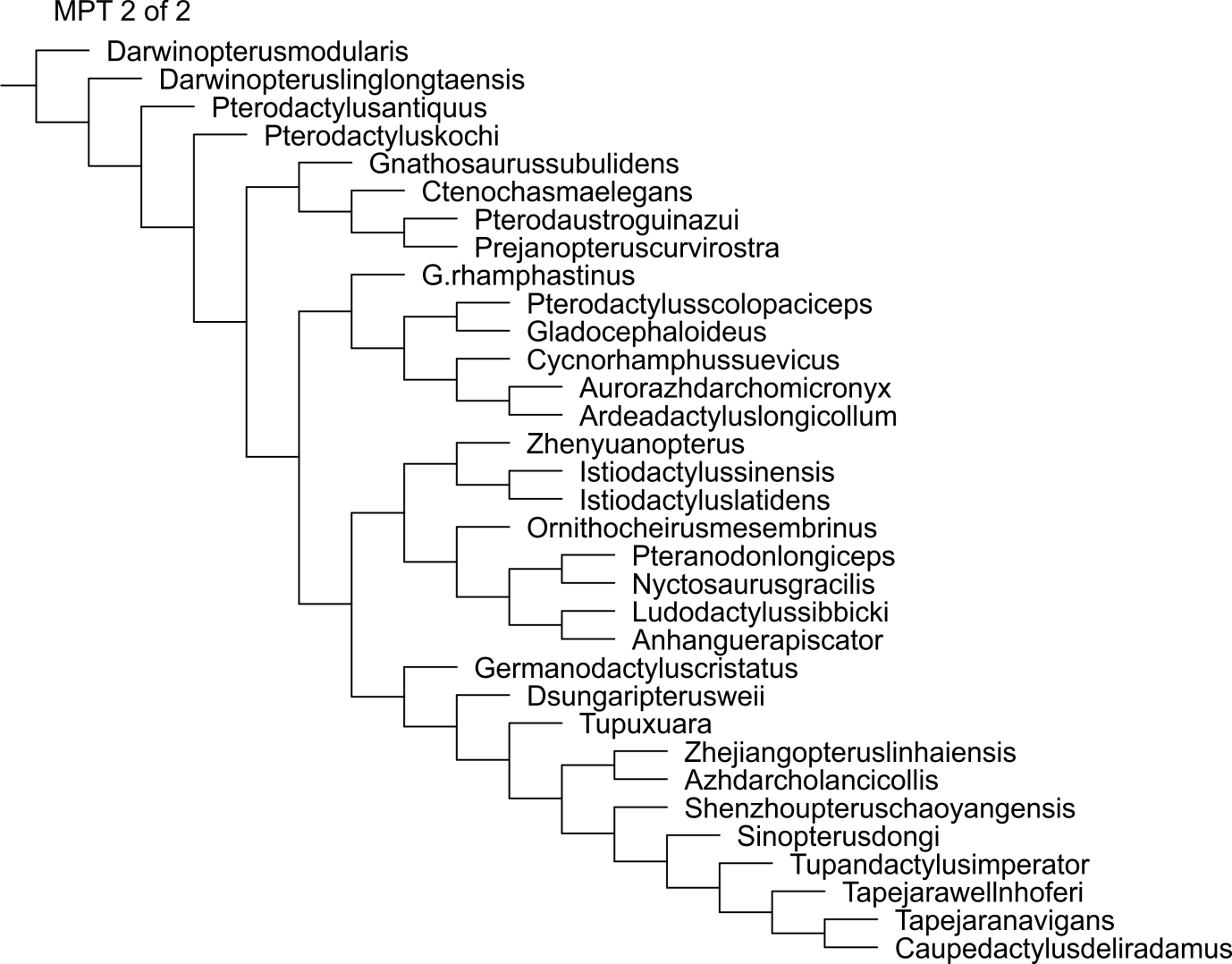


MPT 2 of 2 recovered. The same topology was resolved in both search functions (i.e. NT search and Traditional).


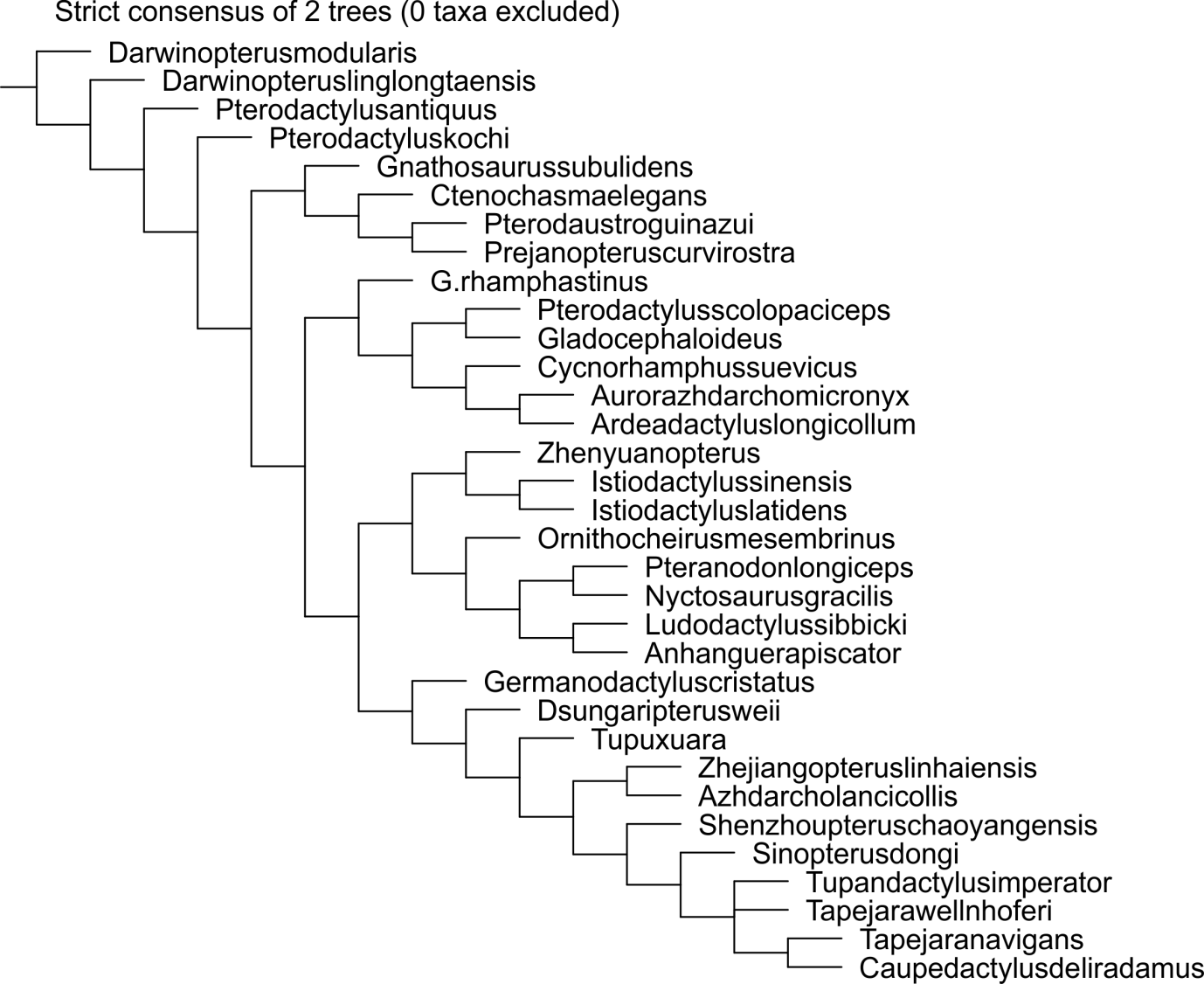


Strict consensus of two MPTs.

**S1.4.1.**

BSP AS XIX
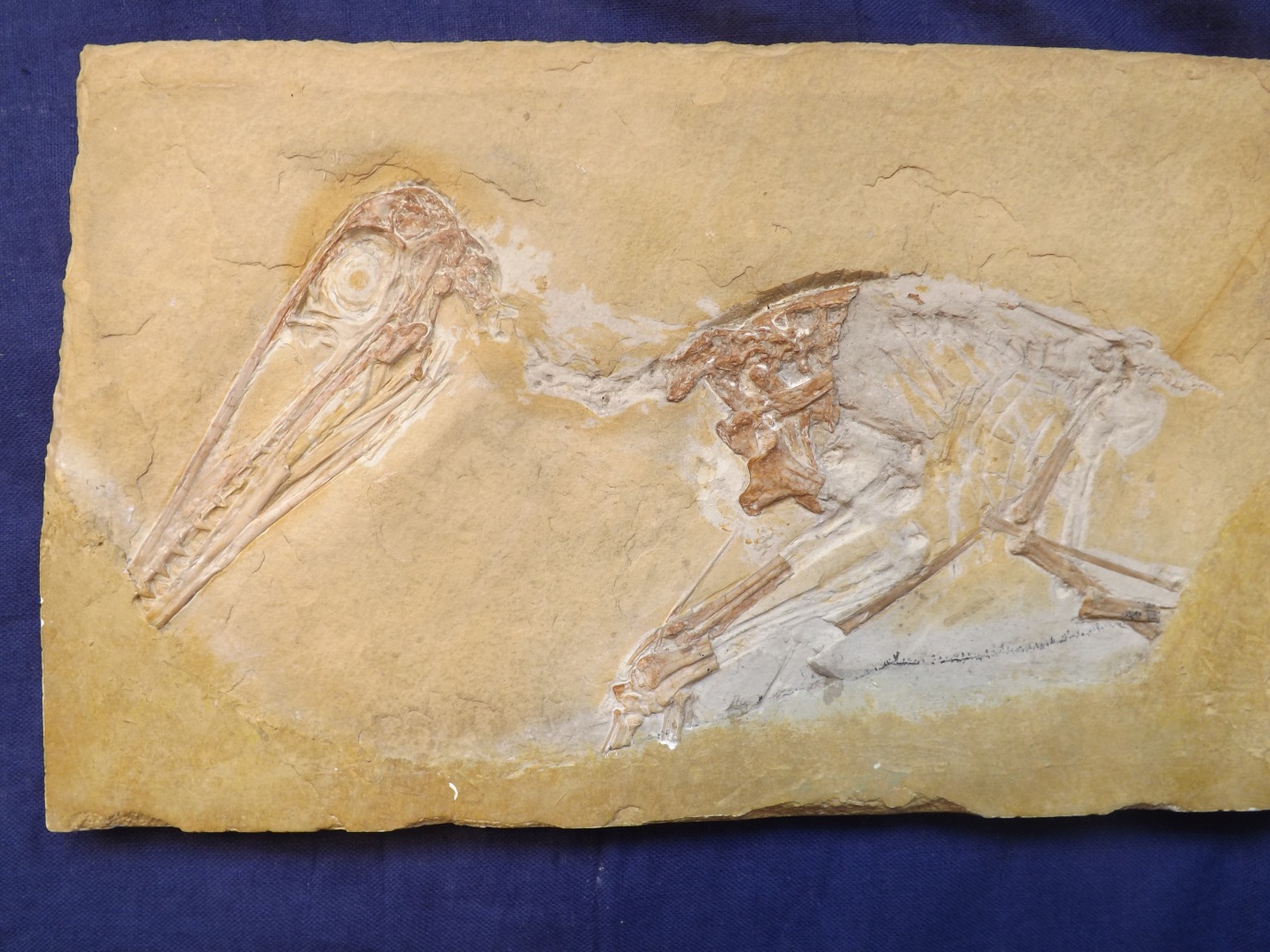


SMF R 404
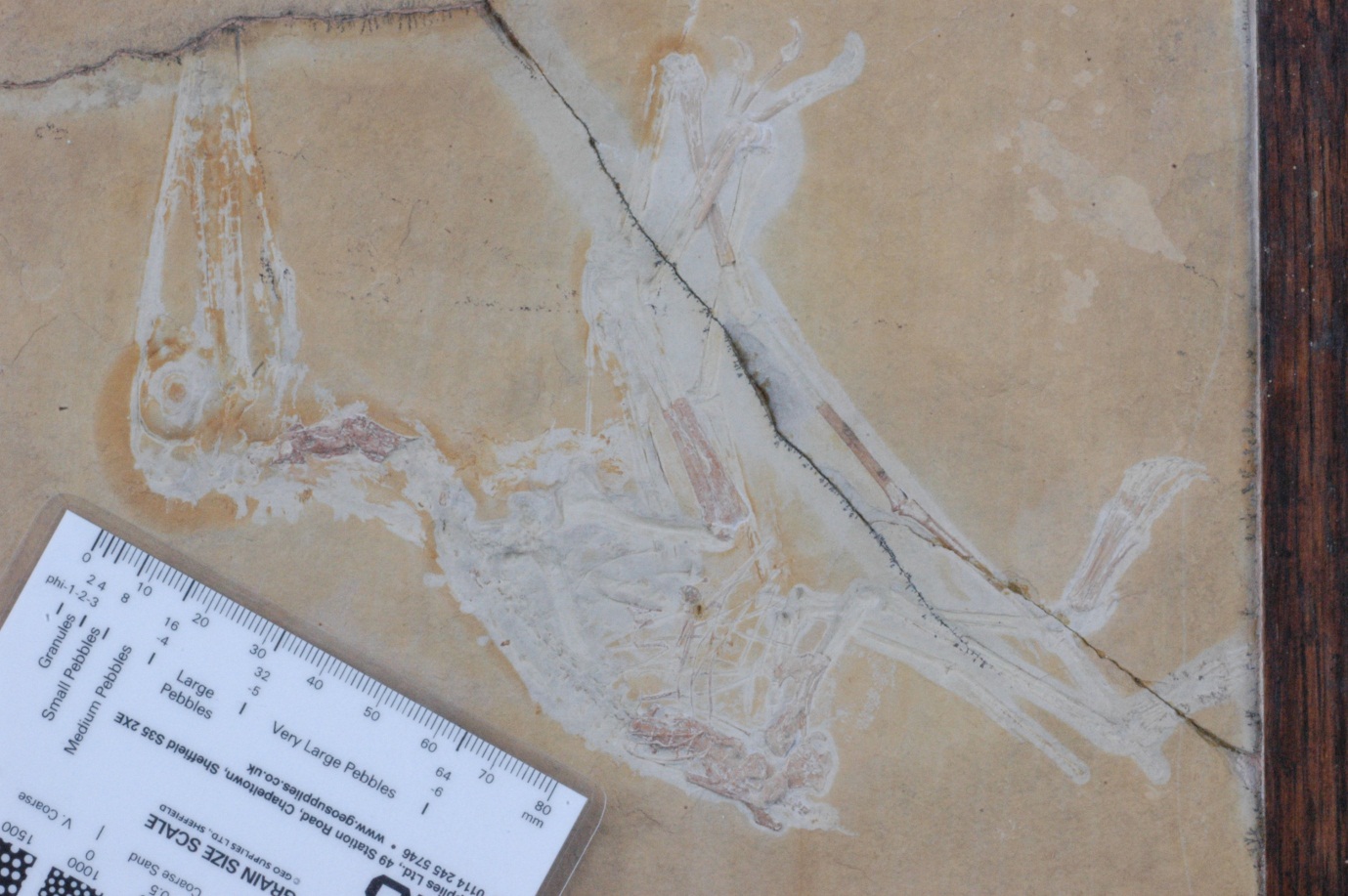


SMF R 4072
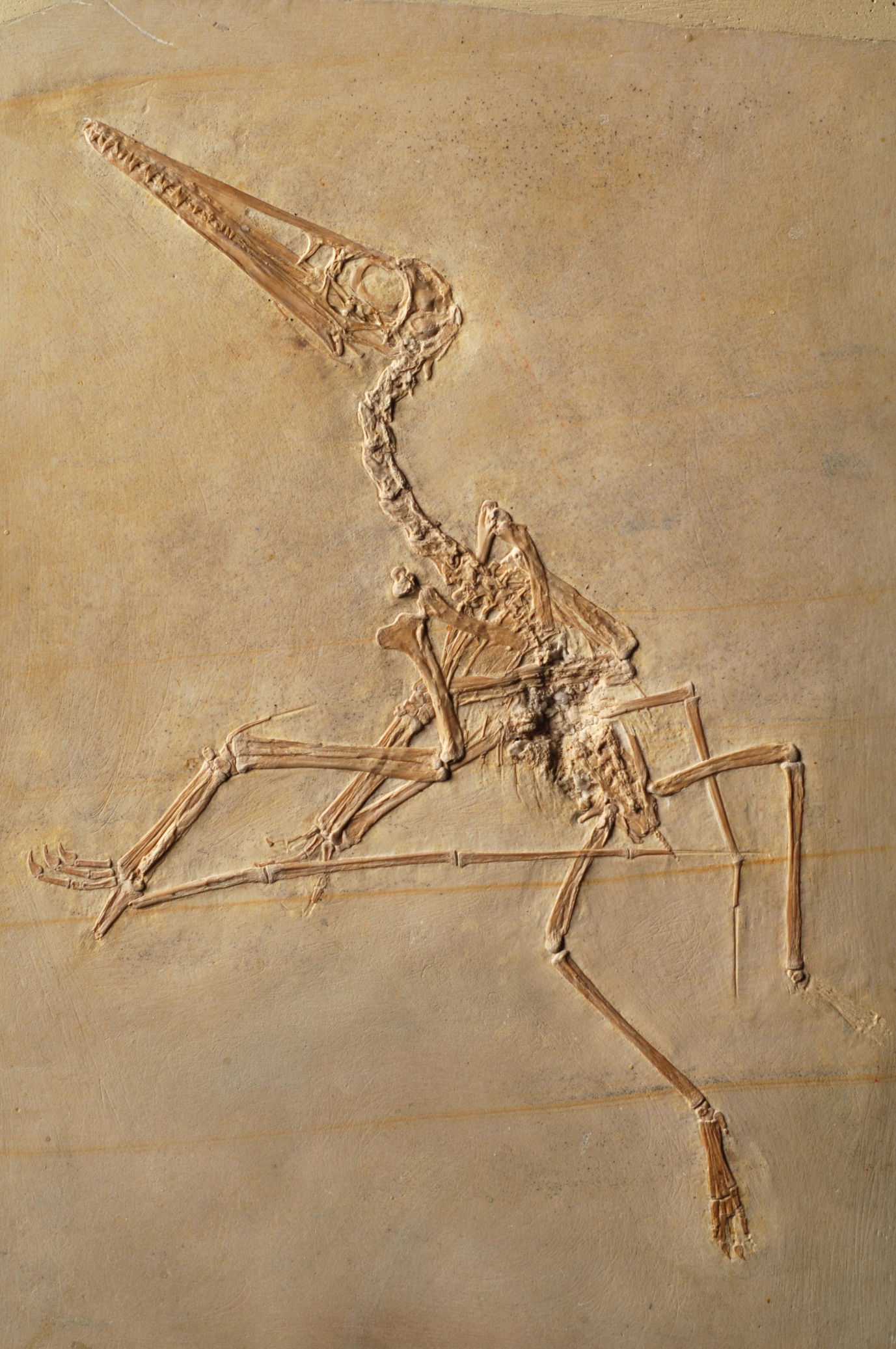
BSP AS I 739
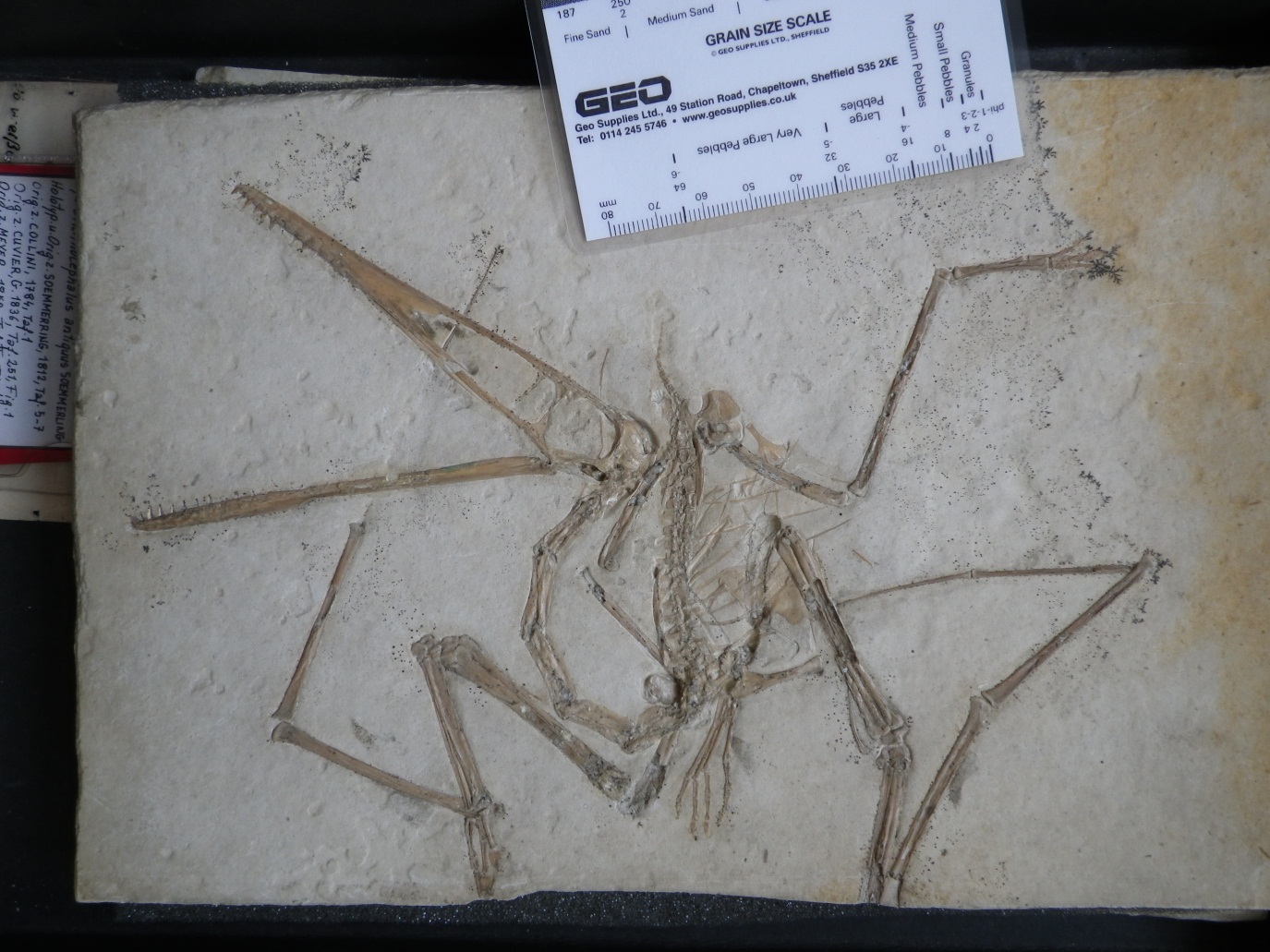


BSP 1883 XVI 1
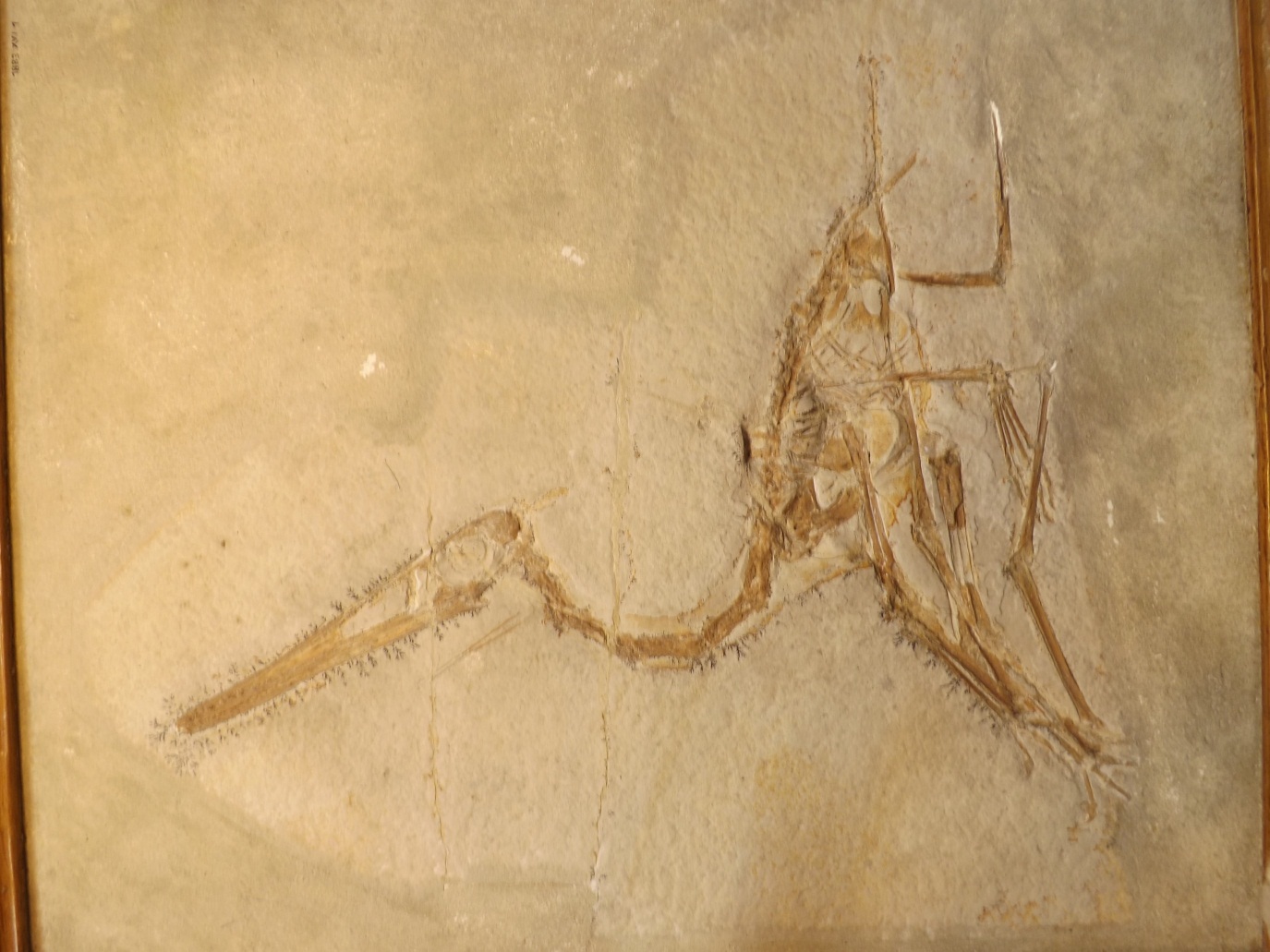


BSP 1975 I 221


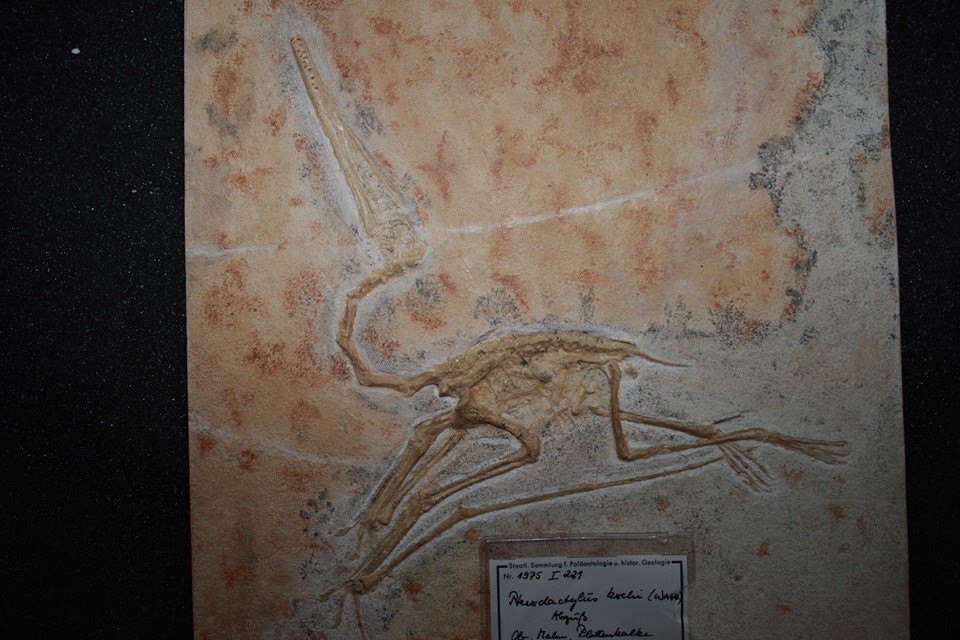


NHMUK PV R 3949
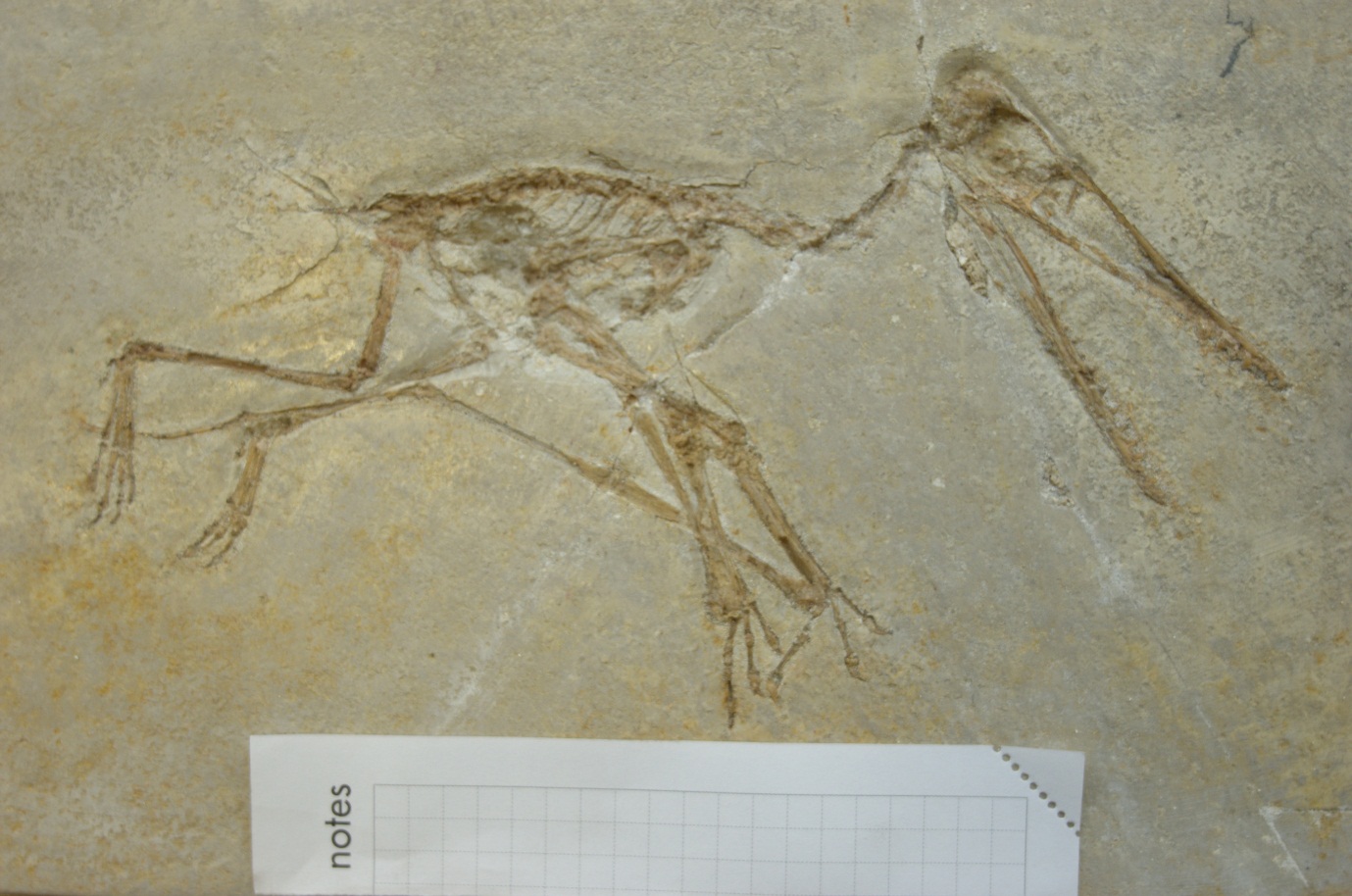


OUMNH JZ 1609
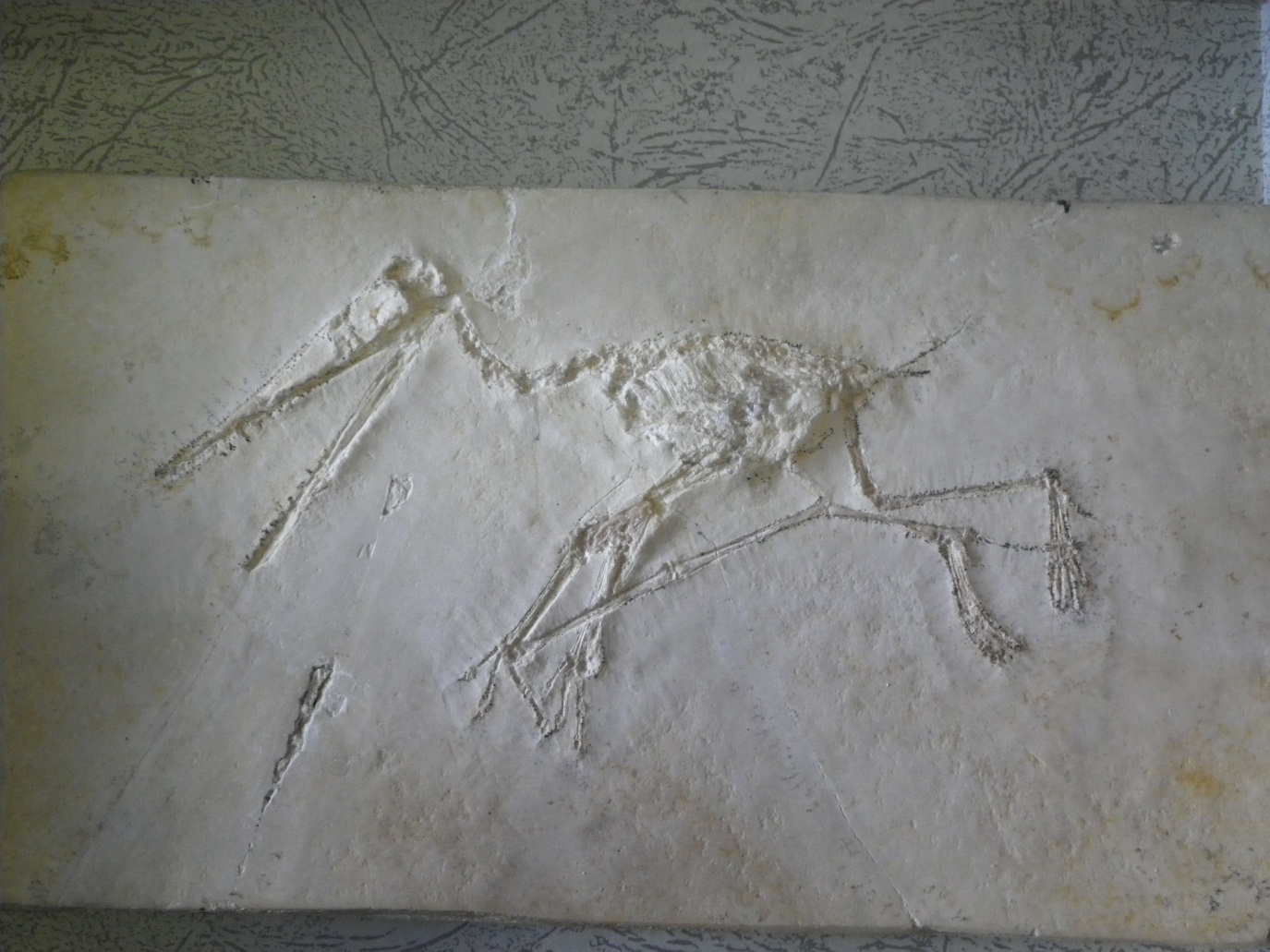

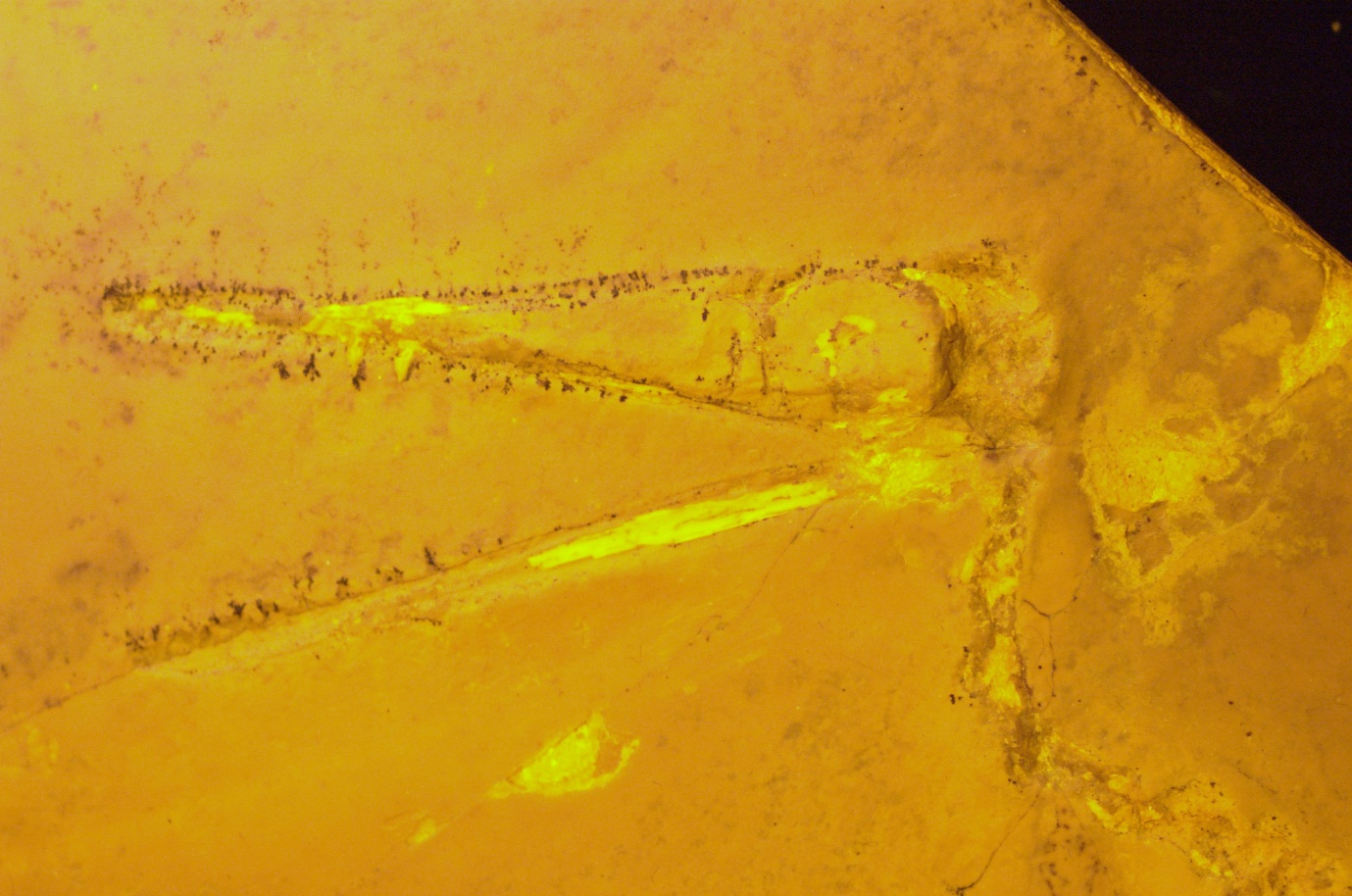


**References**

Brazeau M D (2011) Problematic character coding methods in morphology and their effects. Biological Journal of the Linnean Society 104: 489-498.

Goloboff PA, Farris JS, Nixon KC (2008) TNT, a free program for phylogenetic analysis. Cladistics 24: 774-786.

Lü JC, Unwin DM, Jin X, Liu Y, Ji Q (2010) Evidence for modular evolution in a long-tailed pterosaur with a pterodactyloid skull. Proceedings of the Royal Society B 277: 383-389.

Pereyra V, Mound LA (2009) Phylogenetic relationships within the genus *Cranothrips* (Thysanoptera, Melanthripidae) with consideration of host associations and disjunct distributions within the family. Systematic Entomology 34: 151-161.

Wang X, Kellner AWA, Jiang S, Meng X (2009) An unusual long-tailed pterosaur with elongated neck from western Liaoning of China. Anais de Academia Brasileira de Ciencias 81: 793-812.
